# Discrimination in lexical decision

**DOI:** 10.1371/journal.pone.0171935

**Published:** 2017-02-24

**Authors:** Petar Milin, Laurie Beth Feldman, Michael Ramscar, Peter Hendrix, R. Harald Baayen

**Affiliations:** 1 Department of Journalism Studies, The University of Sheffield, Sheffield, United Kingdom; 2 Haskins Laboratories and Department of Psychology, SUNY, Albany, United States of America; 3 Seminar für Sprachwissenschaft, Eberhard Karls Universität Tübingen, Tübingen, Germany; Rijksuniversiteit Groningen, NETHERLANDS

## Abstract

In this study we present a novel set of discrimination-based indicators of language processing derived from Naive Discriminative Learning (ndl) theory. We compare the effectiveness of these new measures with classical lexical-distributional measures—in particular, frequency counts and form similarity measures—to predict lexical decision latencies when a complete morphological segmentation of masked primes is or is not possible. Data derive from a re-analysis of a large subset of decision latencies from the English Lexicon Project, as well as from the results of two new masked priming studies. Results demonstrate the superiority of discrimination-based predictors over lexical-distributional predictors alone, across both the simple and primed lexical decision tasks. Comparable priming after masked corner and cornea type primes, across two experiments, fails to support early obligatory segmentation into morphemes as predicted by the morpho-orthographic account of reading. Results fit well with ndl theory, which, in conformity with Word and Paradigm theory, rejects the morpheme as a relevant unit of analysis. Furthermore, results indicate that readers with greater spelling proficiency and larger vocabularies make better use of orthographic priors and handle lexical competition more efficiently.

## 1 Introduction

The mental lexicon is typically conceptualized as a repository of lexical representations, hierarchically organized into networks of units varying in granularity, including phonemes, syllables, and words in auditory comprehension, and letters, letter n-grams, rhymes, morphemes, and words in reading [1–10]. Opinions differ with respect to the computational architecture and the computational processes operating on these representations. These have been described alternatively with mechanisms based on symbolic rules, interactive activation, spreading activation, or Bayesian probability theory. The general consensus is that word forms exist in the mind, and that various counts of their properties and constellations underlie how they are processed by the mind. Similarity among word forms is captured by neighborhood counts or by in-degrees in lexical graphs. In what follows, we refer to these kinds of measures as lexical-distributional variables. Contributions of frequency of occurrence, neighborhood density, and length in letters can be complemented with many other predictors, such as counts of synonyms and counts of morphologically related words, letter or phoneme n-gram frequencies, and syllable frequencies [11]. Counts can also be refined in various ways. For instance, counts of neighbors can be weighted for proximity or interconnectedness within similarity neighborhoods [12, 13].

Count measures—whether counts of neighbors or straightforward counts of occurrences—have proved quite useful for predicting lexical decision reaction times and accuracy. However, the use of counts linked to lexical units such as words raises the question of how these units are discriminated from other similar units. Models, ranging from the interactive activation model of [14] to the Bayesian Reader of [15], account for the identification of word units by means of a hierarchy of sublexical units, such as letter features and letters, and algorithms sorting out the evidence provided by lower-level units for higher-level units.

A very different approach is explored by [16]. They constructed a two-layer network with weights estimated by application of the simplest possible error-driven learning rule, originally proposed by [17]. As input units they used letter pairs, and as output units, semantic units. In what follows, we refer to these units, which are pointers to locations in a high-dimensional semantic vector space [18–22], as *lexomes*. What [16] observed is that the network, when trained on 20 million words from the British National Corpus, produced activations for target words that shared many properties with observed reaction times in the visual lexical decision task. For instance, reaction times are predictable to a considerable extent from measures such as whole word frequency, constituent frequency, family size, and inflectional entropy, and the same holds for the activations produced by the network. Remarkably, even relative effect sizes were closely matched between empirical and simulated reaction times.

Crucial to understanding the predictions of error-driven learning is to realize that while correct prediction leads to strengthening of the associations (weights) between features (henceforth cues) and discriminated categories (henceforth outcomes), misprediction results in weakened association strength. [23] provides a telling example from vision. When a visual prime (a picture of a grand piano) precedes a target stimulus (a picture of a table) which has to be named, naming times are delayed compared to unrelated prime pictures. This phenomenen, named anti-priming by Marsolek, arises as a consequence of error-driven learning. When recognizing the grand piano, weights from features such as “having legs” and “having a large flat horizontal surface” are strengthened to the grand piano, but at the same time weakened to “table”, even though tables have legs and large flat surfaces. Precisely because these cues have just been downgraded as valid cues for tables after having recognized a grand piano, subsequent interpreting and naming the picture of a table takes more time. This example illustrates the continuous tug of war between input cues competing for outcome categories—a tug of war that resists precise quantification by means of simple counts. Crucially, the association strength of a given cue to a given outcome is co-determined not only by how often this cue and this outcome co-occurs, but also by how often this cue co-occurs with other outcomes. This important insight is taken into account by, e.g., the statistics for two-by-two contingency tables proposed by [24–26].

The intricacies of error-driven learning, however, are not well captured even by these high-level statistical measures. This is because the association strength between a cue *c*_*i*_ and an outcome *o*_*j*_ not only depends on how often *c*_*i*_ co-occurs with other outcomes, but also on how often other cues *c*_*j*_ that are present in the visual input together with *c*_*i*_ co-occur with other cues and other outcomes. This continuous between-cue calibration across learning histories gives rise to, e.g., phenomena such as the secondary family size effect reported by [27]. As a consequence, a proper estimate of the weight on the connection from *c*_*i*_ and *o*_*j*_ actually depends on the complete history of events in which *c*_*i*_ was present and weights were adjusted as a function of whether outcomes were predicted correctly or incorrectly. Thus, this approach characterizes the mental lexicon as a dynamic system in which seemingly unrelated constellations of cues far back in learning history can have unexpected consequences for current processing.

A first goal of the present study is to examine in further detail whether measures derived from principles of discrimination learning might outperform classical measures based on counts. In this endeavor, we depart from the previous study by Baayen et al. (2011) in several ways.

First, as cues, we use letter trigrams instead of letter bigrams, as we have found that for English this systematically gives rise to more precise prediction. For other languages, letter bigrams may outperform letter trigrams, see [28] for the case of Vietnamese.

Second, we extend the activation measure of the previous study with several new network statistics, and test their predictive value for unprimed and primed lexical decision times. Below, we present these new additional measures in further detail.

Third, the original model of Baayen et al. (2011) was in fact a decompositional model at the level of lexomes. It did not include lexomes for complex words such as *works*, *worker*, and *workforce*, and posited that the evidence for a complex word is obtained by integrating over the evidences for its constituents. However, subsequent work [28, 29] has shown that excellent predictions are obtained when complex words are entered into the model with their own lexomes. From a semantic perspective, this has the advantage of doing justice to the often unpredictable shades of meanings of complex words. For instance, English *worker*, although often described in theoretical treatises as meaning ‘someone who works’, in actual language use is found to denote someone employed for manual or industrial labor, to denote a member of the working class, or to denote a usually sterile member of a colony of bees that carries out most of the labor. It turns out that both whole-word and constituent frequency effects emerge for free when complex words are granted their own lexomes [28]. Furthermore, since morpheme frequency effects emerged in the network of Pham and Baayen in the absence of form representations for morphemes, the revised model fits well with recent approaches in theoretical morphology such as word and paradigm morphology [30], which eschew the morpheme as theoretical construct.

This brings us to the second goal of the present study, the vexed issue of blind morpho-orthographic segmentation in visual word recognition. According to [31], it is the orthographic and not the semantic properties of morphemes that have profound consequences early in visual word recognition. Rastle and colleagues argue that the visual system parses *corner* into *corn* and *er*, even though *corner* is semantically unrelated to *corn*. Conversely, for a word like *brothel*, segmentation into *broth* and *el* is said not to take place. Even though *el* is a frequent letter sequence in English, appearing in words such as *level, angel, personnel, apparel, wheel, barrel, jewel*, and many others, it is not a true morpheme of this language. Different priming outcomes for prime-target pairs such as *corner–corn* and *brothel–broth* have been taken as support for the importance to the earliest stages of visual processing of the morpheme-as-form, a purely orthographic unit devoid of semantics.

The theory of morpho-orthographic segmentation is incompatible with linguistic theories such as word and paradigm morphology, and it is also incompatible with discriminative learning theory. The present study confronts these opposing perspectives on reading with data from both unprimed and primed lexical decision. Generalized additive mixed models (gamms) with predictors grounded in discrimination learning are compared with gamms using classical lexical distributional covariates. In what follows, we first discuss the issues surrounding the theory of morpho-orthographic segmentation. We then introduce in more detail our theory of discrimination learning and associated measures, after which we proceed to discuss experimental data from unprimed and primed lexical decision.

### 1.1 Morpho-orthographic segmentation

In the third volume of his monumental ‘The art of computer programming’, Knuth suggested that an algorithm of dictionary look-up might be made more efficient by stripping of prefixes, and looking up prefixed words in separate lists indexed by their stems [32]. If the stripping off of prefixes proceeds automatically purely on the basis of an initial form-based match (e.g., initial *re* matching *redo*), words such as *reindeer* are split up into *re* and *indeer*. While from an engineering perspective, one can add *indeer* to the list of stems to make look-up more efficient, Taft and Forster, in a series of publications [1, 33, 34] pursued the hypothesis that semantically incomplete forms such as *indeer* are not available as entry points in human lexical access. In the absence of such entry points, prefix stripping is predicted to give rise to a processing cost, since after failure to locate an entry point for *indeer*, a new search for the original full form, *reindeer*, has to be initiated. Processing delays observed across several experiments for pseudo-prefixed words such as *reindeer* were taken as evidence for semantically blind segmentation and automatic form-driven stripping of prefixes.

However, an investigation of the lexical statistics of prefixes and pseudoprefixed words reported by [35] revealed that instances of pseudo-prefixed words were so common token-wise that no advantage of prefix stripping is to be expected in a serial look-up architecture. The problem is that although pseudo-prefixed words are relatively uncommon type-wise, they tend to have very high token frequencies of occurrence. If automatic segmentation were to take place, and if *ach* (as in *reach*) is not available as access point, the gain in efficiency obtained by stripping *re* of *redo* is offset by a loss of efficiency incurred by time and again having to backtrack from stripping off prefixes in words such as *reach, reindeer, read, ready, dear, debit, uncle*, and *under*. Therefore, if prefix stripping would indeed be characteristic of human lexical access, it would brand human lexical access as ineffecient and extremely far removed from the ideal of Bayesian rationality.

The line of research pursued [31, 36] proposes that suffixed words undergo obligatory suffix stripping. The evidence for this hypothesis concerns pseudo-suffixed words such as *corner* and form-similar controls such as *brothel*. Pseudo-suffixed words such as *corner* can be parsed into the independently existing noun *corn* and *er* (cf. for prefixed words *debit*, which can be decomposed into *de* and *bit*), even though semantically this parse makes no sense. Controls such as *brothel* contain the embedded word *broth*, but since *el* is not a suffix of English, Rastle and Davis argue that *brothel* is not decomposed. Several primed lexical decision experiments have been reported for which average reaction times to the stem were comparable for truly suffixed primes (*worker*) and pseudo-suffixed primes (*corner*), whereas control primes (*brothel*) were found not to prime the corresponding onset-aligned embedded words (*broth*).

From an algorithmic perspective, however, similar problems arise as in the case of prefix stripping. For instance, a perusal of the celex lexical database [37] shows that in 57% of all English words containing the terminal letter pair *er*, the letter sequence *er* does not function as a suffix.

To complicate matters further, it is not clear why it would be advantageous to separate forms in *er* from their stems. The problem is that *er* does not have a single semantic function, but instead expresses a wide range of meanings, including comparison (*greater*), agency (*walker*), instrument (*opener*), causation (*howler*), and patiency (*shooter*, as in *this bear is a shooter*; see [38, 39]). Furthermore, *er* is also found in words that are clearly subject (agent) nouns, but are not derived from any particular base words; for example, *father*, *mother* and *brother*, which fit in with the category of persons denoted by *er* in agent nouns such as *baker* and *butcher*, or *buyer* or *seller* (but do not fit the category of comparatives). This semantic fragmentation of *er* and the token-wise preponderance of pseudosuffixed words with *er* such as *her*, diminish the utility of a purely orthographic form representation for *er*.

This problem is aggravated by the semantic idiosyncrasies of many derived words (cf. the example of *worker* mentioned above). In order to know what a suffixed word means, stem and affix need to be considered jointly. Pulling them apart is counterproductive for interpretation, while rendering lexical access less efficient computationally. The question thus arises whether morpho-orthographic decomposition is actually taking place.

There are several reasons for considering this issue in further detail. First, the experimental evidence is not straightforward. [40] did not replicate morpho-orthographic effects from masked priming in another task, the same-different task. [41] and [42] report experiments indicating an early processing disadvantage for pseudo-complex words such as *corner*. They call attention to the nonword materials, and point out that the kind of nonwords used may induce subjects to follow different strategies while carrying out lexicality decisions in the masked priming task. [16] expressed concerns about stimuli such as *fruitless* and *archer*, classified as pseudo-complex by [31], but which are both etymologically derived and synchronically much more transparent to their base words compared to words such as *corner*. [43] replicated early morpho-orthographic effects for English with stimuli that are semantically better controlled. In this study, however, pseudo-suffixed primes had significantly larger orthographic neighborhoods than suffixed primes (*t* = 2.039, *p* = 0.0239), a difference this study did not bring into the statistical analysis.

Second, the studies reporting evidence for morpho-orthographic segmentation make use of a between-items design in which target words differ across priming conditions (cf. *worker*, *corner* and *brothel* as masked primes for *work*, *corn*, and *broth*). This raises the question of what the outcome would be when a statistically more powerful within-items design is used. One possible outcome is stronger support for morpho-orthographic segmentation, and the evaporation of evidence for early semantic processing. Alternatively, stricter statistical control might reallocate variance due to having different target words across priming conditions, resulting in lack of support for early morpho-orthographic segmentation.

Third, statistical analyses of data addressing morpho-orthographic segmentation appear to have systematically neglected the prime as a repeating stimulus. Priming effects are accounted for only by means of factorial contrasts (priming condition). The fact that the same prime occurs in, e.g., a suffixed condition and the unrelated benchmark condition is not brought into the statistical analysis. This comes with the risk of the analysis being anti-conservative.

Finally, it is worth considering the evidence for morpho-orthographic segmentation when traditional lexical-distributional predictors are replaced by measures grounded in learning theory. Learning-based measures take into account the extent to which letter sequences such as er# (with the # representing the space character) support the lexomes of words such as *corner* and *worker*. A decompositional account would stipulate a-priori that the connection strength from the low-level visual unit er# to the lexomes *worker* and *corner* could be important as the motor for morpho-orthographic segmentation, while the connection strength from *el#* to *brothel*, *personnel* or *barbel* would be assumed to be irrelevant and to all practical purposes be very close to zero. Learning-based measures make it possible to assess this kind of predictions empirically.

In the present study, we begin with inspecting unprimed lexical decision latencies from the English Lexicon Project [44], and show that they outperform classical lexical-distributional measures. We then report two masked priming experiments addressing the issue of early obligatory morpho-orthographic segmentation, which we also analyse with both lexical-distributional predictors and learning-based predictors. Both experiments make use of a within-targets design, while bringing prime word into the statistical analysis as random-effect factor. Before discussing these experiments and analyses, however, we first introduce the learning theory, and the measures derived using this learning theory, that we will use as predictors in our statistical models.

## 2 Grounding predictors in discrimination learning

### 2.1 The Rescorla-Wagner equations

Our learning measures build on a theory of learning that is anchored in the equations for error-driven discrimination learning proposed by [17]. These equations implement the simplest possible learning rule in which prediction error plays a central role. The Rescorla-Wagner rule is related mathematically to the perceptron of [45], but it is simpler in that there is no sigmoid squashing function to normalize total activation at output units. It is also related to the learning rule of [46], which specifies how regression weights can be updated incrementally as input becomes available over time.

Several studies on language acquisition have shown that error-driven learning, and the Rescorla-Wagner model in particular, adequately predict human behaviour across various language-related tasks and problems [26, 47–50]. Furthermore, the anti-priming effects observed by [23] fit well with the Rescorla-Wagner equations. The Rescorla-Wagner equations have a strong following in the animal learning literature [51, 52], and evolutionary simulation studies suggest that, in all its simplicity, this learning rule may have advantages over more complicated, and theoretically more precise, alternative learning regimes [53]. In sum, the Rescorla-Wagner learning rule has been considered among biologically the most plausible of all learning algorithms [54]. In this study, however, we will use these equations primarily to provide a functional and computationally tractable characterization of human error-driven discrimination learning.

Formally, a two-layer discrimination network can be described as follows. Let C denote the complete set of input units, henceforth *cues*, with cardinality *k*, and let O denote the set of all possible output units, henceforth *outcomes*, with cardinality *n*. After exposure to all training data, a Rescorla-Wagner network will be defined by a *k* × *n* weight matrix. During learning, the actual weight matrix will be smaller, as typically only subsets of cues and outcomes will have been encountered at a given point in learning time. Weights are adjusted for each learning event *L*_*t*_, *t* = 1, 2, …, *T*. At a given learning event *L*_*t*_, weights are adjusted on the connections from the cues actually present in the input of that learning event, henceforth the *active* cues $C_{t}$ ($C_{t}\subseteq C$), to all of the outcomes *o*_1,…,*t*_ that have been encountered at least once during any of the learning events *L*_1_, *L*_2_, …, *L*_*t*_. Let the set of outcomes present at learning event *L*_*t*_ be denoted by $O_{t}$ ($O_{t}\subseteq O$). The weight $w_{ij}^{\left(t\right)}$ from cue $c_{i}\in C_{t}$ to outcome $o_{j}\in O_{t}$ after *t* learning events is given by

$$\begin{array}{c} w_{ij}^{\left(t\right)}=w_{ij}^{\left(t-1\right)}+\Delta w_{ij}^{\left(t-1\right)}. \end{array}$$ (1)

The change in the weight, $\Delta w_{ij}^{\left(t-1\right)}$, is defined by the Rescorla-Wagner equations:

$$\begin{array}{c} \Delta w_{ij}^{\left(t-1\right)}=\{\begin{array}{cc} 0 & \text{if}\;c_{i}\notin C_{t},\\ \alpha_{i}\beta_{j}(\lambda -\sum_{m}{\text{I}}_{\left[c_{m}\in C_{t}\right]}w_{mj}^{\left(t-1\right)}) & \text{if}\;c_{i}\in C_{t}\wedge \;o_{j}\in O_{j},\\ \alpha_{i}\beta_{j}(0-\sum_{m}{\text{I}}_{\left[c_{m}\in C_{t}\right]}w_{mj}^{\left(t-1\right)}) & \text{if}\;c_{i}\in C_{t}\wedge \;o_{j}\notin O_{j}\wedge o_{j}\in O_{1,\ldots ,t-1},\\ 0 & \text{otherwise}. \end{array} \end{array}$$ (2)

In our calculations, *λ* is set to 1.0 and *α*_*i*_ = *β*_*j*_ = 0.1. The product *α*_*i*_ *β*_*j*_ = 0.01 defines the learning rate, *λ* represents the upper limit of learning. These equations specify that if the *i*-th cue is not one of the active cues at learning event *L*_*t*_, none of its efferent weights are changed. If the *i*-th cue is one of the active cues at learning event *t*, the connection to outcome *j* is strengthened if this outcome is also present at *L*_*t*_. If this outcome is not present, but has been encountered during some previous learning event, its weight is adjusted downward. The amount by which weights are adjusted is determined by the other active cues and their weights to the pertinent outcome. The sum of these weights is subtracted from the maximum learning potential *λ* when outcome *j* is present, and from zero when it is not. Thus, in the presence of many cues, positive adjustments tend to be small and negative adjustments large.

Although two-layer networks have been claimed to be incapable of solving linearly non-separable classification problems [55], we have found two-layer networks trained with the Rescorla-Wagner equations to provide excellent non-linear separation, provided that an appropriate representation is available for the input units.

**Fig 1:**
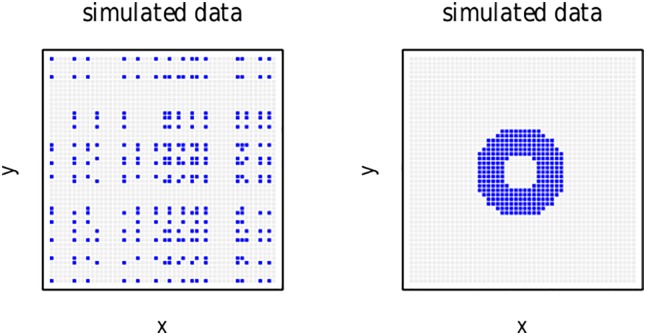
Non-linear pattern of simulated data. Left panel: original simulated data. Right panel: re-arranged simulated data.

To appreciate the potential of Rescorla-Wagner networks, consider, as an example of a non-linearly separable classification problem, the left panel of Fig 1, which presents a 50 × 50 grid of datapoints, of which 260 (highlighted in blue) are to be classified as *A*, and the remaining 2240 (in gray) are to be classified as *B*. Each data point is described by a triplet (*x*, *y*, *r*), where *r* is the corresponding class label (*A* or *B*). When *x* and *y* are defined as coordinates in a Cartesian grid, there is no straight line that separates the classes *A* and *B*. In other words, given standard coordinates in $R^{2}$, the two classes are not separable. However, we can reformulate this classification problem in a higher-dimensional space by taking *x* and *y* to be binary labels *x*_1_, *x*_2_, …, *x*_50_ and *y*_1_, *y*_2_, …, *y*_50_, with *x*_*i*_, *y*_*j*_ ∈ {0, 1}, that specify whether a point is located on some specific column (or row) in the matrix. Importantly, since *x* and *y* are labels, rather than integers or reals, we can re-arrange the *x*_*i*_ and *y*_*j*_ labels without changing the classification problem. One such re-arrangement gives rise to the circular pattern for the *A* class presented in blue in the right panel of Fig 1. Although this re-arrangment separates the two groups spatially, they still cannot be separated by a straight line.

**Fig 2:**
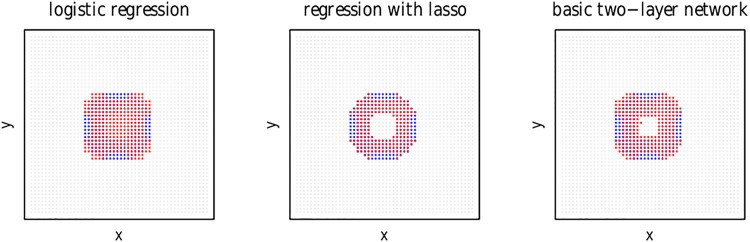
The results of three ‘linear’ classification algorithms applied to the simulated data, using 1-hot encoding for column and row membership. Left panel: standard logistic regression, middle panel: logistic regression with lasso regularization, right panel: standard Rescorla-Wagner learning. Red pixels indicate predicted class A responses, blue pixels indicate true class A responses.

As can be seen in the left panel of Fig 2, a standard logistic regression model (glm) predicting class (*A*: 1, *B*: 0) from the indicator variables *x*_1,2,…,50_ and *y*_1,2,…,50_ captures a majority of the data points of class *A*, but fails to include points at the edges, while incorrectly including points in the center. (Equivalently, a glm can be fitted to factors *X* and *Y*, with each factor having 50 levels, one for each column or row of the data grid.) Shrinking of the *β* coefficients of a glm using lasso (ℓ1-norm) regularization as implemented in version 2.0-2 of the glmnet package for r (run with *maxit* = 300) [56] correctly assigns all points in the inner disk to class *B*, but otherwise fails in the same way at the edges, as shown in the middle panel of Fig 2.

The right panel of Fig 2 shows results obtained with a Rescorla-Wagner two-layer network, classifying the 260 most active data points as class *A*. With a single training cycle, this simple network improves upon the standard glm by correctly assigning most data points in the center to class *B*, and yields a pattern of results that is qualitatively similar to that of a logistic glm with lasso regularization.

A 3-layer backpropagation network with 4 hidden layer units learns to classify the data without error. This neural network was implemented through the deeplearning() function of the h2o package in r [57] and was presented with the input data 500 times. A gradient boosting machine (gbm) [58, 59] fit with 20 trees with a maximum tree depth of 20 using the xgboost package for r, version 0.4-3 [60] and a support vector machine with a second order polynomial kernel (fit using the svm() function in version 1.6-7 of the e1071 package [61] for r) were equally successful.

Interestingly, the performance of a two-layer network can be brought up to the same level, at least for this data set, as that of state-of-the-art techniques in machine learning through two pairs of input units that are sensitive to the local environment. The first pair of units specifies whether or not all neighbors of a given datapoint (in the representation of the data in the right panel of Fig 1) belong to class *A*. We refer to these datapoints as hubs. The second feature encodes whether or not any of the neighbors of a datapoint is a hub. Using only these two pairs of two input features (is hub, is not a hub, is neighbor of hub, is not neighbor of a hub), a two-layer network yields perfect classification performance in a single training cycle.

These results clarify that two-layer networks are much more powerful than previously thought. A basic out-of-the-box two-layer Rescorla-Wagner network already achieves considerable success in non-linear separation, comparable to that of a glm with lasso shrinkage. Enriching the model with input units that are sensitive to the local environment offers a boost in performance that, although computationally light, allows for perfect class separation with only a single pass through the data.

The crucial point is that the performance of a ‘model’ is determined not only by the algorithm used (backpropagation or the Rescorla-Wagner learning rule), but also by the representations on which these algorithms operate. The present classification example cannot be solved when representations are integers or reals in a Cartesian grid. When representations are chosen in a 100-dimensional indicator space, the classification problem can be solved by some techniques, whereas other techniques offer good, albeit not perfect, ‘non-linear’ solutions. When smart representations are selected that characterize the classes in a local receptive field, the classification problem can be solved completely by a two-layer network with only four input units, with minimal computational costs.

In what follows, we used the Rescorla-Wagner equations two build two discrimination networks. The first network, henceforth the G2L network, takes letter trigrams as cues (input units), and has lexomes as outcomes (output units). The second network, henceforth the L2L network, has lexomes both as cues and as outcomes. First consider the G2L network.

### 2.2 The G2L network

The G2L network takes graphemic units as inputs and semantic units (lexomes) as outputs. We selected letter triplets as cues, with the space character denoted by #. Thus, the first letter triplet of *corner* is #co and the last letter triplet is er#.

The output units are lexomes, defined as pointers to locations in a high-dimensional semantic vector space. Such spaces can be generated from WordNet [62] as in the study of [63]. Semantic vectors can also be generated from co-occurrence statistics, as in Latent Semantic Analysis [18], Hyperspace Analogue of Language [20, 21], or the Topics model [22]. More recently, prediction-driven approaches have shown excellent performance as well [64]. The second discrimination network (L2L) discussed below sets up a semantic vector space by applying the Rescorla-Wagner equations to predict lexomes from neighboring lexomes. For the G2L network, the output units are pointers to words’ locations in this vector space.

Three aspects of this approach are noteworthy. First, unlike [63], we do not set up a network to learn a mapping between a vector of letter trigrams and a semantic vector. Instead, the G2L network is designed to discriminate, on the basis of sublexical orthographic cues, between lexomes.

Second, it is well known from the categorization literature that categories are in constant flux, as apparent already from the example of anti-priming discussed above [23, 65, 66]. The theoretical construct of the lexome—we use a neologism here to avoid misunderstanding with well-established terms such as ‘lemma’, ‘lexeme’, or ‘meaning’—provides us with the computational flexibility to work with fixed references to semantic vectors while at the same time allowing the content of these semantic vectors to evolve as experience with the language accumulates.

Third, lexomes do not stand in a one-to-one relation to orthographic words. This is clarified by the examples in Table 1. (For further discussion of idioms in discrimination learning, see [67]). Three of the many expressions in English for *dying* are listed first, together with a subset of trigram cues and an index pointing to an arbitrary numbered semantic vector that represents dying in a semantic vector space. Even though the word forms are very different across the three expressions, they share the same lexome. Conversely, homographs such as *bank* have different lexomes.

**Table 1:** Example cues and outcomes for synonyms and homographs.

forms	cues	outcomes
pass away	#pa, pas, ass, s#a, …	387
kick the bucket	#ki, kic, ick, k#t, …	387
die	#di, die, ie#	387
apple pie	#ap, app, ppl, ple, …	4
take money from the bank	#ta, tak, ake, e#m, …	87620, …
the bank of the river	#th, the, he#, e#b, …	2031, …

The index for ‘dying’ is 387, the index for bank as financial institution is 87620, the index for bank as the solid edge of a river is 2031, and that for apple pie is 4.

**Fig 3:**
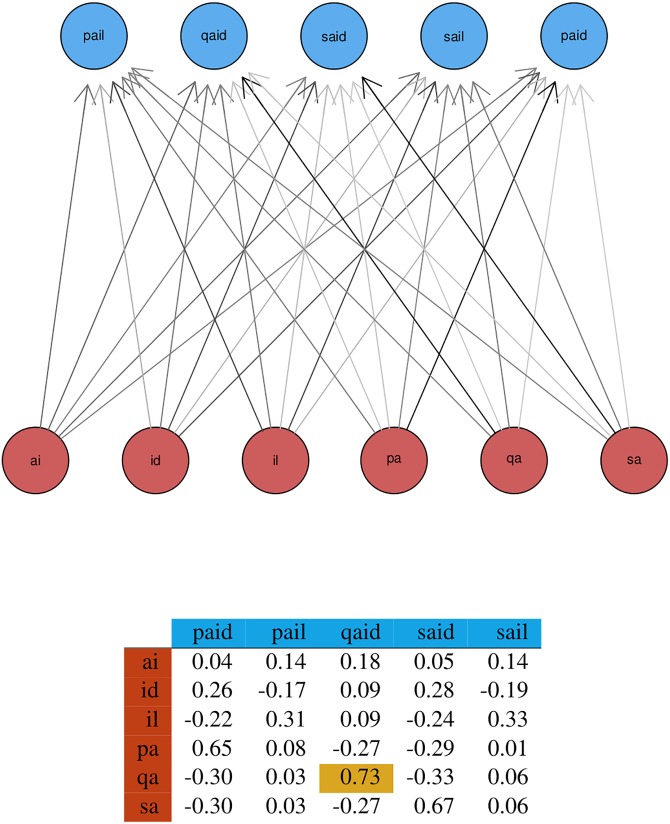
Layout of a Rescorla-Wagner network. Network layout (top) and weight matrix (bottom), obtained with the equilibrium equations of [68] for the Rescorla-Wagner equations, for the lexomes *paid, pail, qaid, said, sail* presented with frequencies 550, 50, 1, 9900, and 50, using letter bigrams as orthographic cues.

Fig 3 illustrates a G2L network for a simple example with a set of lexome outcomes O={paid,pail,qaid,said,sail} (we use words instead of numbers as lexomic indices to facilitate interpretation) and a set of orthographic bigram cues C={ai,id,il,pa,qa,sa}. (For ease of exposition, the space character is not taken into account in this example; the weights shown here were calculated with the equilibrium equations for the Rescorla-Wagner equations given by [68], which capture the endstate of learning.) The weight from the bigram *qa* to the legal scrabble word *qaid* (tribal chieftain) is highlighted, as it illustrates that *qa* is a highly discriminative cue. Being unique to *qaid*, it supports this lexome, while suppressing *paid* and *said*, at the same time leaving less similar words (*sail, pail*) unaffected.

### 2.3 The L2L network

Above, we defined the lexome as a pointer to a location in a high-dimensional co-occurrence based semantic vector space. The lexome-to-lexome (L2L) network sets up such a vector space.

[69] constructed, using the English child-directed speech in childes, a lexome-to-lexome (L2L) network by moving a 5-word window through the childes corpus and taking the center word as outcome and the two flanking words on either side as cues. Cosine similarities calculated between row vectors of this L2L matrix captured semantic similarities between a large number of different categories of nouns (months of the year, weather words, drinks, tools, etc.), with better precision than semantic vectors obtained by applying a range of standard techniques such as Latent Semantic Analysis [18], Hyperspace Analogue of Language [20, 21], and the Topics Model [22] to the same data.

Findings such as these suggest that row vectors in the L2L matrix are indeed semantic vectors. A crucial property of these semantic vectors is that they are not static, i.e., as learning progresses, these vectors are subject to continuous updating, and hence are highly dependent on the experience accumulated up to a given point in developmental time. This dynamicity is, we believe, a highly desirable property of semantic vectors. Some recent vector-based semantic models are, actually, developing in the direction of incremental updating (see, for example, the hal-based approach of [70], and the word2vec model of [64]).

The weight matrix of the L2L network constructed for the present study is derived from the bnc instead of from childes. A three-word window was moved across the corpus, and for each window, the center word was the outcome while the flanking words constituted the cues. For the semantic categories studied by [69], we re-calculated the average within-category cosine similarity as well as the average cosine similarity with the words outside that category. For 5 out of 29 categories used in the above-mentioned study, *t*-tests revealed no significant difference (*p* > 0.05) between the two group means, for the remaining categories, differences were well supported (*p* < 0.001, for details, see Table 2; a sign test indicates that under randomness, it is highly unlikely to obtain 24 out of 29 hits, *p* = 0.0003). This result confirms that the row vectors of our L2L weight matrix are indeed interpretable as semantic vectors, and that results are relatively independent of the corpus selected for training and the details of the window used.

**Table 2:** Semantic categories, difference of mean within group and outside-group similarity, *p*-value, and number of words in a category, using the similarity matrix derived from the L2L weight matrix estimated from the British National Corpus.

Category	Difference	*p*-value	number of words
dessert	-0.040	0.050	12
times	-0.031	0.384	11
plants	-0.026	0.157	13
bathroom	0.032	0.051	19
clothing	0.036	*p* < 0.001	37
games	0.042	0.257	6
toys	0.060	*p* < 0.001	23
bird	0.065	*p* < 0.001	22
meat	0.066	*p* < 0.001	15
fruit	0.069	*p* < 0.001	19
body	0.075	*p* < 0.001	64
tools	0.077	*p* < 0.001	24
space	0.098	0.002	12
numbers	0.102	*p* < 0.001	27
insect	0.110	*p* < 0.001	15
mammal	0.112	*p* < 0.001	52
drink	0.116	*p* < 0.001	14
kitchen	0.120	*p* < 0.001	25
family	0.122	*p* < 0.001	33
electronics	0.127	0.000	17
shape	0.129	*p* < 0.001	12
days	0.130	*p* < 0.001	14
weather	0.138	*p* < 0.001	13
music	0.141	*p* < 0.001	12
vegetable	0.148	*p* < 0.001	15
vehicles	0.168	*p* < 0.001	32
furniture	0.207	*p* < 0.001	25
household	0.247	*p* < 0.001	32
months	0.420	*p* < 0.001	13

### 2.4 Predictors derived from the G2L and L2L networks

Several measures can be calculated from the weight matrix of a discrimination network. For the G2L network, a first measure, studied previously in [16], sums the afferrent weights from a word’s orthographic cues (its active cues at time *t*, $C_{t}$) to its lexome, resulting in that lexome’s activation *a*_*t*_. Thus, for the *j*-th lexome, its activation is given by

$$\begin{array}{c} a_{j}=\sum_{i\in C_{t}}w_{ij}^{\left(t\right)}. \end{array}$$ (3)

For *qaid*, the activation given the weights in Fig 3 is 0.73 + 0.18 + 0.09 = 1.00.

The activations of the lexomes on the output layer of the network, given a set of active input cues, tend to follow a lognormal distribution, i.e., they show a similar rightward skew as reaction time distributions. [16] observed that G2L activations predict reaction times in the visual lexical decision task, with greater activation affording shorter response latencies. They also observed that these activations, when taken as proxies for reaction times, capture a wide range of empirical phenomena, ranging from surface and stem frequency effects to effects of morphological family size [71] and paradigmatic relative entropy [72]. [73]. [74] found lexome activation to be predictive for fixation durations, and [28] reported that these activations explain the otherwise puzzling anti-frequency effect of constituents in Vietnamese visual word recognition.

A given set of active cues produces a vector of activations over the output units. If all activations are close to zero, the input stimulus fails to make contact with the lexomes. As a consequence, the stimulus will not be interpretable, and amounts to nothing more than visual noise. Real words will tend to produce activation vectors with non-zero values that can be either positive or negative. Because of the presence of negative weights, the Shannon entropy is not available as a characteristic of the amount of information (or uncertainty) of the activation vector. Alternatives are provided by the first and second vector norms. Give a vector of activations **a**, the *p*-norm of this vector is given by

$$\begin{array}{c} {\left|\text{a}\right|}_{p}={\left(\sum_{i}{\left|\text{a}\right|}^{p}\right)}^{1/p}. \end{array}$$ (4)

For *p* = 1, we obtain the absolute length of the vector, and for *p* = 2 we obtain its Euclidian length. The median absolute deviation MAD [75, 76] provides a third alternative.

These three measures are all highly correlated. In our experience, the absolute length (1-norm) performs best as a predictor of behavioral response measures. The 1-norm highlights mathematically the extent to which there are lexomes that are relevant given the input. Lexomes that are irrelevant, and that have an activation close to zero, do not contribute to the 1-norm. In what follows, we rely on 1-norm of the activation vector as measure of competition, and refer to it as the G2L activation diversity (a-diversity), as it captures the diversity of co-activated lexomes.

**Fig 4:**
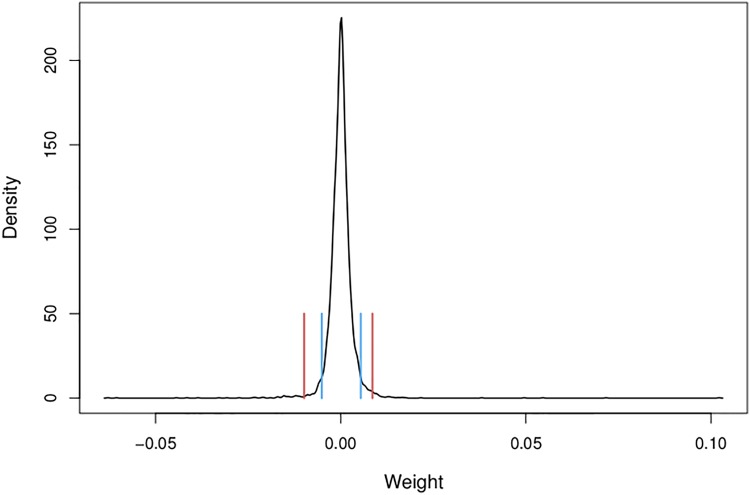
Example probability density of afferrent weights. Estimated probability density for the weights on the incoming links from orthographic trigrams to the lexome corner. Red lines present boundaries for the highest 50 absolute weights, blue lines indicate the boundaries for the 150 highest absolute weights.

The weights in a column (or a row) of a network’s weight matrix are characterized by a spiky distribution, with mode around zero, that can be approximated by a Generalized Hyperbolic distribution or by its special case, the Normal-Inverse Gaussian distribution. Fig 4 provides, by way of example, the estimated probability density function for the column vector of *corner* in the weight matrix of the G2L network. The vertical lines highlight the tail areas with 150 and 50 large positive or negative weights respectively. In other words, there are relatively few weights of large absolute magnitude, whereas most of the probability mass of the weights is concentrated around zero.

It follows from the spiky shape of the distribution of row and column weights that the weight matrix of a discrimination network is not dense but sparse. This sparseness is of interest not only because it reduces the effective complexity of the model, but also because it fits well with the known sparseness in neuronal connectivity [77] and the relatively low numbers of different signals to which mtl cells are responsive (50—150 according to the estimates of [78]). Note that although our model is a functional model and not a neuro-biological model, we find it interesting that the relatively low number of nonzero weights on effective afferent or efferent connections apparently mirrors actual sparsity in real neurobiological networks.

Geometrically, the column vectors of the G2L matrix give the coordinates of lexomes in a multidimensional orthographic space. In order to obtain numeric characterizations of their locations in this orthographic space, we can apply the absolute and Euclidean distances as well as the median absolute deviation to these column vectors. Again, the three measures (mad, 1-norm, and 2-norm) are highly correlated, and as before, the 1-norm emerged from our investigations as slightly superior to the other two. Importantly, the column 1-norm is a systemic lexome measure that is independent of what cues are actually active in the input. Hence, it functions as a measure of the prior availability of a lexome, or, to use a metaphor, its network entrenchment. We henceforth refer to this measure as the G2L prior.

The 1-norm of a column vector in a discrimination network’s weight matrix has a ‘mechanistic’ interpretation. Both neurons and neuronal assemblies are known to have baseline firing rates that are modulated as they engage with experienced stimuli [79–81]. By analogy, in our artificial discrimination network, we can allow cues to propagate activation to lexomes, proportional to the connection weights, also in the absence of visual input, albeit to a much reduced extent. As a consequence of this baseline firing of cues, outcomes receive continuous bottom-up support, resulting in baseline activations similar to the resting activation levels familiar from multilayer connectionist and interactive activation models. These baseline activations also resemble priors in Bayesian models of visual and auditory comprehension [7, 15]. However, whereas their Bayesian priors are based on straightforward frequency counts, normed into probabilities, column 1-norms, albeit strongly correlated with frequency of occurrence, provide a measure that presupposes a role for discrimination when evaluating the consequences of frequency of occurrence in experience.

The G2L prior (column 1-norm) has proven useful as a predictor of decision latencies and eye-movement behavior. [29] showed that the G2L prior is a strong predictor of lexical decision latencies as well as of age of acquisition. For age of acquisition ratings, the G2L 1-norm emerged as a predictor with a greater variable importance in a random forest analysis than measures of written and spoken frequency. Furthermore, [74], in an analysis of the eye-movement record for the reading of compounds in continuous discourse, found that whereas the G2L activation was predictive for initial fixations, the G2L prior emerged as a key predictor for second fixation durations.

In summary, from the G2L matrix, we derived three measures: the target lexome’s activation (representing its bottom-up support), the activation diversity (representing the amount of uncertainty produced on the output layer given the input stimulus), and the G2L prior, a lexome’s availability independent of any input.

Four further measures can be derived from the weight matrix of the L2L network: a lexome’s Semantic Density, a lexome’s semantic typicality, a lexome’s prior availability, and a lexome’s l-diversity. A lexome’s L2L prior availability parallels G2L prior availability, where the former is obtained by taking the 1-norm from its column vector in the weight matrix of the L2L network. [29] observed that the L2L prior is a strong predictor of lexical decision latencies, outranked only by subtitle frequency in a random forest analysis over their test-case data.

A second measure derived directly from the L2L weight matrix is the 1-norm of a lexome’s row vector. This measure assesses the extent to which other lexomes are co-activated when a given lexome is the input cue. The greater the number of words that are co-activated, the greater the amount of information in the semantic vector, and the longer response latencies are predicted to be. In what follows, we refer to this measure as the L2L l-diversity, in parallel to the G2L a-diversity measure.

The remaining two measures are based on the cosine similarity matrix calculated from the L2L weight matrix. From a *k* × *n* L2L weight matrix, with *k* input and *n* outcome lexomes, a new and symmetric *k* × *k* matrix can be derived that for each pair of lexomes provides their semantic similarity gauged by applying the cosine similarity metric to the *n*-element row vectors of these lexomes in the L2L weight matrix. A lexome’s semantic density is obtained by calculating the number of other lexomes with a cosine similarity exceeding 0.9. Our expectation is that a high semantic density will render a lexome more ambiguous, and hence longer response latencies should be characteristic of higher values of semantic density (see also [19, 21]).

We can also ask to what extent a given semantic vector is similar to the average semantic vector. Greater similarities are an index of the semantic ‘typicality’ or the ‘unremarkableness’ of a lexome. A higher value of semantic typicality is therefore expected to predict shorter response latencies.

A final predictor that we found useful is a word’s written frequency (in the British National Corpus) residualized on the above learning-based predictors. Our knowledge of concepts and categories is determined not only by the frequencies with which their word labels are mentioned in speech or writing, but also by our interaction with the world. Although we expect that language frequency and world-frequency are correlated, they are unlikely to be identical. The residualized frequency measure, henceforth concept frequency, is an attempt to estimate the part of frequency that cannot be traced to discriminative learning, and that we hypothesize to provide a window, albeit imperfect, on the learning that allows conceptual categories to be discriminated on the basis of non-linguistic cues [23, 65, 66].

All predictors with a rightward skew in their distributions were log-transformed in order to facilitate statistical analysis. The same procedure was applied to lexical-distributional predictors.

### 2.5 Estimation of weight matrices

A 1.1 billion word corpus of English subtitles [82] was used for estimating the G2L matrix. Previous studies [83] suggest that subtitles are an excellent choice for modeling response latencies in the visual lexical decision task, albeit in all likelihood because short and emotionally charged words are used in this particular genre to a much greater extent than in normal spoken or written language [29, 84]. Letter trigrams were used as orthographic cues, and space-separated letter sequences were used as indices for semantic vectors and constituted the lexomes. We opted for this corpus primarily for its size, with the aim of obtaining good estimates of the weights from letter n-grams to lexomes.

For the L2L weight matrix, we chose the British National Corpus [85]. Unlike subtitle corpora, which typically comprise shortened versions of scripted speech, the British National Corpus provides a balanced sample of English registers. Although much smaller (100 million words), we decided to give greater weight to quality over quantity for the semantic vector space.

In the next section, we compare classical lexical-distributional measures with the above discrimination-based measures as predictors for the (unprimed) lexical decision latencies of the English Lexicon Project [44].

## 3 The English Lexicon Project lexical decision latencies

[11] analyzed the by-item mean lexical decision latencies available from the English Lexicon Project [44], restricting themselves to those available for the subset of the younger subjects. In what follows, we investigate the reaction times of both the younger and older subsets of participants, but confining the analysis to those 1812 words for which all discrimination statistics are available to us.

We transformed the reaction times using the reciprocal transformation -1000/*RT*. The Box-Cox transformation test indicated a reciprocal transformation to be optimal. We changed sign so that coefficients would have the same signs as for models fitted to the untransformed latencies. Multiplication by 1000 was applied in order to avoid extremely small numerical values.

Whereas [11] made use of a linear model, we relaxed the linearity assumption and fitted a generalized additive model [86] to the data. By allowing nonlinear interactions between two pairs of numeric predictors, the fit of the model improved significantly.

### 3.1 Analysis with lexical-distributional predictors

Table 3 summarizes our model with lexical-distributional variables as predictors. Words beginning with a voiceless segment were responded to more quickly than words with other initial segments. PC1 is a measure of orthographic consistency, contrasting forward enemies (number of words with different pronunciation for the same sequence of letters) with phonological neighbors (number of words that differ by a single phoneme), see [11] for further details. Reaction times in lexical decision were slower for words with large phonological neighborhoods than for words with many feedforward enemies. A greater mean bigram frequency predicted longer RTs, young subjects responded faster, and words that are used more often as a noun than as a verb were likewise responded to more quickly. Neighborhood density showed a small inhibitory effect, and verbs were responded to more quickly than nouns.

**Table 3:** Generalized additive model fitted to simplex words from the English Lexicon Project using classical lexical-distributional predictors.

A. parametric coefficients	Estimate	Std. Error	t-value	p-value
Intercept	-1.4122	0.0257	-55.0493	< 0.0001
Voicing = voiceless	-0.0104	0.0036	-2.8625	0.0042
PC1	0.0031	0.0009	3.3240	0.0009
Mean Bigram Frequency	0.0118	0.0029	4.0479	0.0001
Age Subject = young	-0.3224	0.0035	-93.3435	< 0.0001
Noun-Verb Ratio	-0.0018	0.0008	-2.2737	0.0230
N-count	0.0009	0.0004	2.1693	0.0301
Word Category = verb	-0.0119	0.0051	-2.3314	0.0198
B. smooth terms	edf	Ref.df	F-value	p-value
te(Frequency, Family Size): Age Subject = old	7.6470	9.5128	52.2674	< 0.0001
te(Frequency, Family Size): Age Subject = young	10.0868	12.1805	88.0158	< 0.0001
s(Written to Spoken Frequency Ratio)	5.3045	6.5217	8.1968	< 0.0001
te(Inflectional Entropy, Number Complex Synsets	3.0014	3.0028	29.1233	< 0.0001

te: tensor product smooth, s: thin plate regression spline smooth. (AIC = −6185.3; -ML = −3097.7; R-sq. (adj) = 0.749)

**Fig 5:**
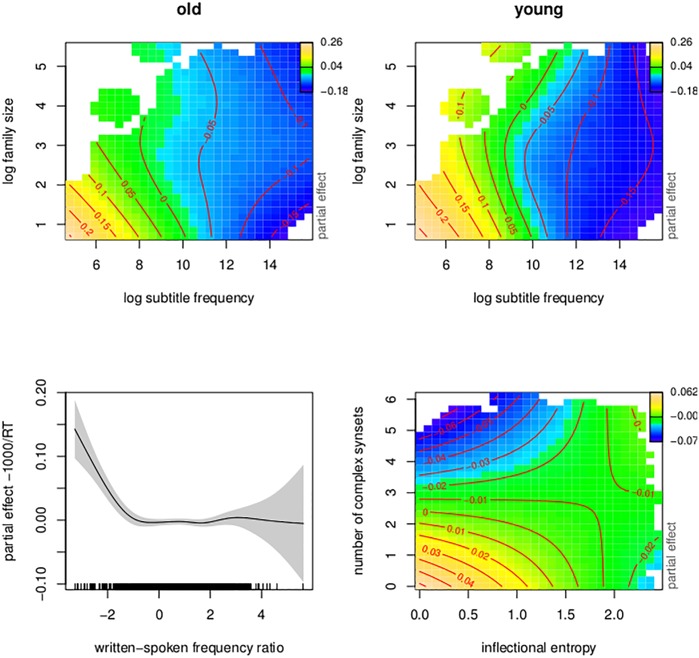
Regression spline smooths for classical predictors. Smooths in the generalized additive model fitted to the visual lexical decision latencies (English Lexicon Project) using classic lexical-distributional predictors.

To understand the smooth terms of the model, visualization is essential. Fig 5 presents the four nonlinear terms of the model, using contour plots. Warmer colors represent longer response times, whereas darker colors indicate shorter response latencies. Contour lines connect points with the same partial effect.

The top panels portray the three-way interaction of frequency, family size, and age. For both young and old subjects, log subtitle frequency (calculated from the abovementioned 1.1 gigaword film subtitle corpus [82]) was facilitatory, and somewhat more pronounced in younger subjects. For both age groups, the effect was attenuated by morphological family size (the number of complex words in which a given target word occurs as a morphological constituent, see [71, 87] for details on this measure). Conversely, the effect of morphological family size was strongest for low-frequency words. For the young subject group, the family size effect was somewhat U-shaped for lower-frequency words.

The lower left panel suggests that words that appear more frequently in speech as compared to writing are processed more slowly. This finding is opposite to what [11] observed, and below we justify why there is reason not to take this pattern seriously. The lower right panel presents the interaction of inflectional entropy, indicating a given word’s paradigmatic complexity [88, 89], and the number of morphologically complex synsets (sets of synonyms) listed for a word in WordNet [62]. For words with few complex synsets, the effect of inflectional entropy is facilitatory, but the effect reverses as the number of synsets increases.

### 3.2 Analysis with learning-based predictors

**Fig 6:**
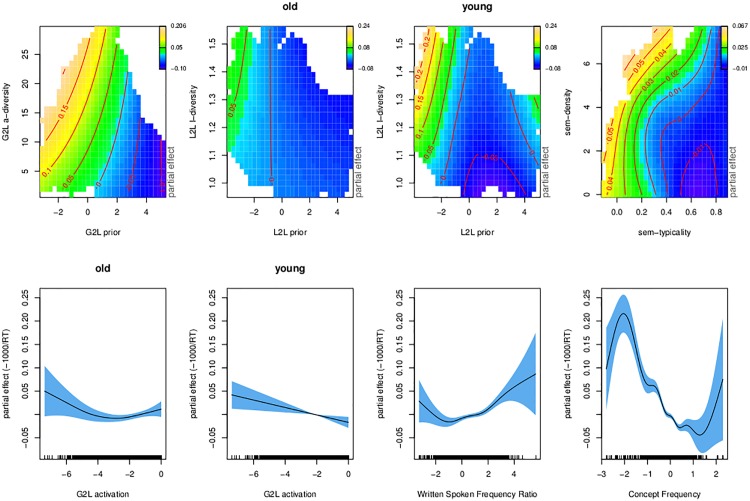
Regression spline smooths for discrimination-based predictors. Bivariate smooths (upper panels) and univariate smooths (lower panels) in the generalized additive model fitted to the lexical decision data from the English Lexicon Project, using discrimination-based predictors.

Table 4 and Fig 6 summarize the generalized additive model fitted to the lexical decision latencies with our learning-based predictors.

**Table 4:** Generalized additive model fitted to simplex words from the English Lexicon Project using discrimination-based predictors.

A. parametric coefficients	Estimate	Std. Error	t-value	p-value
Intercept	-1.3186	0.0024	-543.1538	< 0.0001
Age = young	-0.3224	0.0034	-93.9030	< 0.0001
B. smooth terms	edf	Ref.df	F-value	p-value
te(G2L prior, G2L a-diversity)	3.0008	3.0016	44.5369	< 0.0001
te(L2L prior, L2L l-diversity): Age = old	4.5693	5.3621	5.3898	< 0.0001
te(L2L prior, L2L l-diversity): Age = young	6.9382	8.3329	17.1926	< 0.0001
te(sem-typicality, sem-density)	7.1147	8.8957	5.4650	< 0.0001
s(G2L activation): AgeSubject = old	2.4596	3.1492	2.6914	0.0430
s(G2L activation): AgeSubject = young	1.0003	1.0005	8.0608	0.0045
s(Written Spoken Frequency Ratio)	4.2019	5.2811	8.1942	< 0.0001
s(Concept Frequency)	8.1445	8.8031	27.3368	< 0.0001

te: tensor product smooth, s: thin plate regression spline smooth. (AIC = −6220.1; -ML = −3106.6; R-sq. (adj) = 0.752)

The upper left panel of Fig 6 presents the interaction of the G2L prior and the G2L activation diversity. As expected, a stronger prior affords shorter response latencies. Conversely, the greater the activation diversity, i.e., the more different lexomes have strong activation support, the slower responses become. The effect of the prior is stronger for words with high G2L activation diversity (abbreviated as: a-diversity); in the graph, the horizontal gradient is greater for large G2L a-diversity values than for small values (and contour lines are closer together). At the same time, there is little or no effect of G2L a-diversity for large G2L priors (for which the contour lines are nearly vertical). When the prior is large and the a-diversity is small, it is likely that the target lexome is the only one with good support bottom-up. In the absence of uncertainty due to other lexomes also receiving strong activation, responses can be fast (lower right corner of panel [1, 1]). Conversely, responses are slow when the prior is small and many other lexomes are well activated (upper left corner in panel [1, 1]).

The two upper central panels of Fig 6 present the three-way interaction of L2L prior, L2L l-diversity, and age. First consider the panel for the older participants ([1, 2]). Here, we see a very mild effect of the prior, with an initial downward gradient that starts to level off at about one third of its range. This effect of the prior remains almost unmodulated by the effect of lexome diversity. For the lowest priors, we can see a hint of additional inhibition of lexome diversity. For higher priors, the effect of lexome diversity is not present.

In the response pattern of the young participants there is a strong interaction of L2L prior by L2L l-diversity ([1, 3]). The young participant group emerges as less proficient in dealing with the uncertainty that comes with a high L2L l-diversity. When a lexome has strong connections to many other lexomes, such that these other lexomes become co-activated, younger readers suffer more than older readers.

The interaction of semantic typicality and semantic density (Fig 6, the rightmost upper panel [1, 4]) indicates that words with typical, non-remarkable (or non-surprising), semantic vector profiles are responded to more quickly. Basically, recognition gets faster as words behave similarly to many other words across contexts. At the same time, the more similar semantic neighbours a lexome has (with a cosine similarity exceeding 0.9), the slower responses become. The effect of semantic density is attenuated and even disappears for lexomes with more typical semantic vectors.

The activation of the target lexome shows the expected facilitatory effect, which for the younger age group appears more robust and straightforwardly linear. The effect of register (spoken versus written language) is now as expected: Words that dominate in writing as compared to speech are responded to more slowly, in line with previous results obtained for this predictor by [11]. The unexpected effect of written-to-spoken frequency ratio observed in the model with lexical-distributional predictors (i.e., longer response latencies for words that are more frequent in speech, as presented in Table 3 and in Fig 5) is possibly a consequence of multicollinearity between many count-related predictors.

Finally, greater non-linguistic experience supporting the lexome affords shorter responses, as expected. This trend is reflected in the central part of the panel ([2, 4]). Low and high values of Concept Frequency are diverging from this trend, but here the data are sparse which is reflected in wide confidence intervals.

### 3.3 Model comparison

The model with classical lexical-distributional predictors achieves an adjusted R-squared of 0.749 and an aic of −6185.3 with 11 predictors (9 lexical-distributional predictors, Voicing, and Age). A discriminative model using 10 predictors (8 discriminative predictors, Age, and register) achieves a higher adjusted R-squared (0.752) and a lower aic (−6220.1). The evidence ratio exp^34.8/2^ = 36,000,000, which expresses the extent to which the model with the smaller aic is likely to provide a more precise approximation of the data, provides substantial support for the model with discrimination-based predictors. A chi-square test on the models’ ml scores, using the compareML function from the itsadug package for R [90], confirms that the discrimination-based model provides a substantially improved fit to the data (*p* = 0.003). (Note, however, that because the models compared are not nested, comparisons are informal.)

Table 5 helps trace commonalities and differences between the two models by listing the larger correlations between the two sets of predictors. The strongest classical predictors (frequency, family size, and synsets), align with the strongest discriminative predictors (the two priors, the diversity measures, and the activation measure). Importantly, the classical measures are ‘distributed’ across the discriminative measures, positively boosting the priors and the activation, and negatively boosting the diversities that make discrimination harder. In the classical model, a greater frequency predicts shorter reaction times. In the discrimination-based model, all five discriminative dimensions that correlate well with frequency have effects that align with the effect of frequency, predicting shorter reaction times where correlations are positive, and longer reaction times where correlations are negative. We note that while the frequencies with which words occur is of paramount importance to word recognition, it makes sense—given the excellent performance of the model with learning-based predictors—to seek to understand the consequences of frequency for lexical processing in terms of a system that is continuously learning to predict its environment, rather than in terms of a system requiring ‘counters in the head’ in the form of constructs such as activation levels, activation thresholds, or Bayesian prior probabilities, derived straightforwardly from counts of frequency of occurrence.

**Table 5:** Main correlations between classic and discriminative variables.

	G2L Prior	L2L Prior	G2L a-div	L2L l-div	G2L act
frequency	0.91	0.84	-0.70	-0.47	0.72
family size	0.55	0.60	-0.41	-0.34	0.38
Complex Synsets	0.47	0.55	-0.36	-0.24	0.35
Bigram Frequency			0.28		-0.32
Inflectional Entropy				-0.17	
N-count			-0.22		
PC1			-0.18		

Particularly novel is that the effects of family size and number of complex synsets, which in the classic framework are understood as evidence for morphological structure in the mental lexicon, are captured implicitly in the present discriminative approach. This result constitutes further evidence that lexical processing may not depend on morphemic access units. Exactly as claimed by word and paradigm morphology [30, 91], morphemes need not be represented explicitly.

Mean Bigram Frequency shows only weak correlations with discrimination-based measures: negative for G2L activation, and positive for G2L activation diversity. In the classical model, a higher bigram frequency predicts longer reaction times. The positive correlation of bigram frequency with G2L a-diversity predicts that the effect of this learning measure should also delay responding, which is exactly what the learning-based gam predicts. The negative correlation of bigram frequency and G2L activation indicates, again correctly, that a greater activation results in shorter responses. Importantly, the discriminative approach makes clear why the effect of bigram frequency must be inhibitory: The more a bigram is shared across lexomes, the less effective it will be as a discriminative cue.

The correlations for the remaining three predictors (Inflectional Entropy, N-count, PC1) are low, and we refrain from further interpretation. We also do not speculate on the small correlations between the two semantic measures (sem-typicality and sem-density) and classical measures.

## 4 Experiment 1: Primed lexical decision with high interference

In the preceding section, we established that lexical measures grounded in discrimination learning outperform classical lexical-distributional predictors. We now proceed to Experiment 1, with the double aim of establishing how well learning based predictors perform for masked priming on the one hand, and of clarifying the potential role of orthographic morphemes on the other hand.

Experiment 1 compares primes whose structure allows decomposition into a word and a suffix (corner can be segmented into corn and er), with primes that are only partially decomposable (cornea contains corn but ea is not a suffix of English) and with primes that are unrelated in form (a control condition). Differences between exhaustively and partially decomposable primes are at the core of claims for morpho-orthographic segmentation. The standard experimental design permits different target words with primes that are fully (corn+er) and partially (corn+ea) segmentable into morphemes [31, 36]. Such designs, however, leave open the possibility that differences between priming conditions are due to differences between targets that have escaped experimental control [42, 92]. We therefore opted for a more powerful design with prime-target triplets in which the same targets appear across all priming conditions. If a morpho-orthographic effect exists, this design is optimal for detecting it.

The theory of morpho-orthographic segmentation predicts faster responses for prime-target pairs in which the prime is exhaustively decomposable, regardless of a word’s lexical-distributional characteristics. Thus, because *er* can be a morpheme but *ea* cannot, *corner* will facilitate *corn* but *cornea* will not. Conversely, from a discriminative perspective, *corn* will be activated to some extent by both *corner* and *cornea*, just as *hat* is activated by *that* [93]. The discriminative account therefore does not anticipate different priming effects for *corner* and *cornea*. We note here that without actual calculation of weights, it is impossible to predict how exactly the tug of war between trigram cues for lexomes will fall out, especially as different prime words typically contain other embedded letter sequences that are also words (*lid* in *pallid*, *den* in *ridden*, *tile* in *textile*, *tan* in *tartan*, *new* in *sinew*, *imp* in *impure*, etc.).

Experimental protocol was approved by the University at Albany, SUNY (Institutional Review Board DHHS FWA 00001970), with Protocol Identification Numbers for approval: 08260 and 13219 (00000590). Participants provided verbal informed consent once the procedure was described. The Albany IRB approved the procedure without written consent because they classified the procedure as comparable to daily life with respect to discomfort and harassment and because the confidentiality of all participants was respected.

### 4.1 Subjects

Students from The University at Albany, State University of New York, participated in partial fulfillment of the course requirements for introductory psychology. There were 170 participants. All were native speakers of English with no known reading or speech disorders and normal or corrected-to-normal vision. None participated in more than one experiment.

According to [94], spelling skills can be a strong predictor of morphological priming [95, 96]. These authors argue that masked form priming is sensitive to both facilitatory form effects and inhibitory lexical competition, and that a unique contribution of spelling ability can be associated selectively to the latter. Less skilled readers would rely more on context (priming effect) than skilled readers [94]. Similarly, [97] showed that inhibition was stronger for less proficient readers when responding on partial morphological structure in non-words.

We therefore estimated subjects’ reading ability by measuring their performance on a spelling from dictation test using a set of 15 words taken from a study by [98]. There was no overlap between the test words and the words used as experimental items in the lexical decision task. Of interest is whether these test scores support the hypothesis that primes similar in form will be especially beneficial to less skilled readers; i.e., that less skilled readers will be more similar to skilled readers in the form similar conditions than in the unrelated prime condition.

Participation in the experiment was voluntary, and all data were stored without personal identifiers. Measurements from the experiment and reading assessment for each participant were linked using a numerical code, that was then used in statistical modeling to mark participant as a random factor.

### 4.2 Items

Forty-two monomorphemic words were selected as critical word targets. Each was paired with three types of semantically unrelated primes. (a) Primes with morphologically exhaustive structure, consisting of possible English stems and affixes. (b) Primes with partial morphological structure, consisting of possible English stems that ended with a sequence of letters that never functions as an affix. (c) Morphologically unrelated primes, always ending with the same sequence of letters as one of the related primes for that target. The initial letter sequence of unrelated primes differed from that of the target.

Three experimental lists were constructed to allow for counterbalancing. In each experimental list, one third of the critical targets was paired with morphologically exhaustive primes, one third with morphologically partial primes and one third with unrelated primes. For example, the target limb was paired with limber (exhaustively structured derivation), limbo (partially structured, without an affix) and gazebo (unrelated). By constructing materials in this way, frequency, neighborhood density and family size of the morphologically simple target were equated across prime types. This differs from typical studies with factorial designs where targets are nested within prime type and target properties are only matched across conditions on the mean.

Table 6 summarizes means and standard deviations for attributes of the 42 critical target items and their primes, which we used to make sure that the prime words across priming conditions were properly matched on classic lexical-distributional dimensions.

**Table 6:** Means and standard deviations for item properties.

Predictor	Target		Prime					
			exhaustive		partial		unrelated	
	m	sd	m	sd	m	sd	m	sd
Length	3.43	(0.50)	5.97	(0.90)	6.00	(1.00)	5.89	(0.86)
Log Frequency (HAL)	9.33	(1.69)	6.31	(2.14)	6.87	(2.28)	6.49	(1.92)
Orthographic Neighbors	15.22	(6.90)	3.46	(5.15)	2.46	(3.72)	2.13	(2.88)
Phonological Neighbors	26.43	(14.36)	8.64	(10.20)	5.34	(10.61)	4.05	(7.78)
Bigram Frequency by Position	1821.78	(938.95)	3606.05	(1404.98)	3197.05	(1184.75)	3050.26	(1487.03)
Bigram Freq at Trough			1151		804			

For statistical evaluation, we considered a wide range of lexical-distributional predictors: word length, number of orthographic neighbors, number of phonological neighbors, average distances to orthographic neighbors (i.e., old), and average distance to phonological neighbors (pld), Brown Corpus’ frequency [99], logarithm of word frequency as reported by the hal Study [100], subtlex frequency per million words [83], number of words in the orthographic neighborhood with a greater frequency, and, finally, the sum of the bigram counts by position. These measures were taken from the English Lexicon Project [44].

The condition number for this highly collinear set of predictors was substantial (*κ* = 105.01). To avoid over-interpretation of individual predictors, we orthogonalized these predictors using principal component analysis (pca). The scores on the principal components are optimally weighted composites of the original set of variables, and well suited for use in regression modeling [101, 102].

The first two principal components, which were the only components to satisfy the Guttman-Kaiser criterion and to reach significance in the analyses reported below, jointly explained some 64% of variance of the full set of 10 original predictors. Table 7 lists the loadings of the predictors on these PCs. The first component captures neighborhood properties. The old and pld neighborhood measures have high positive loadings on PC1, while the orthographic and phonological neighbor counts (OrthoNeigh and PhonoNeigh) have negative loadings. Thus, words with high scores on the first principal component have few neighbors at greater distances. Conversely, words with low scores on the first component have many neighbors in close proximity. The second component (PC2) captures frequency-related variables: Brown Corpus frequency, hal frequency, and subtitle corpora frequency (subtlex) all show up with very high positive loadings, while the sum of the bigram counts by position has a weak negative loading.

**Table 7:** Loadings of predictors on the principal components.

	PC1	PC2
Bigram Frequency By Position	-0.135	-0.286
Frequency in Brown Corpus	0.040	0.468
Higher Frequency Neighbors	-0.295	-0.333
Length in Letters	0.284	-0.246
Log Frequency HAL	-0.088	0.526
OLD	0.462	-0.051
Orthographic Neighborhood Count	-0.499	-0.009
Phonological Neighborhood Count	-0.463	0.019
PLD	0.356	-0.066
Log Subtitle Frequency	0.025	0.493

The word items were complemented with filler items also consisting of prime-target pairs. First, there were 28 unrelated word-word pairs whose structure mirrored that of the related prime-target pairs (rosary—limp, impure—fig). Then, a total of 70 word-nonword pairs were adjoined to the experimental lists. In Experiment 1a word-nonword pairs were constructed so that 60% (42/70) were similar in form (frolic—frol, gurgle – gurg), and 40% (28/70) were not (baldness—romb). Hence, the ratio of form similar word-nonword pairs matched the ratio of word-word pairs. In Experiment 1b all word-nonword pairs were constructed so that they were dissimilar in form (e.g., frolic—gurg). Here form similarity was restricted to word targets.

### 4.3 Design and procedure

Prime type was manipulated within participants and within the target types in the following way: Each participant saw each target only once, and all of the targets were preceded by exhaustively decomposable, partially decomposable and morphologically unrelated primes, in equal proportion across participants. Additionally, the form similarity ratio for word-word and word-nonword pairs was manipulated between participants, by their random assignment to either Experiment 1a or Experiment 1b.

Each trial began with a 500 ms fixation mark (‘+’), that appeared in the middle of the screen. An isi of 50 ms occurred before the forward mask (a sequence of ‘#’ signs matched to prime length), which lasted for 450 ms. The prime then appeared in lowercase letters for 48 ms at the position of the mask. The target was printed in uppercase letters and replaced the prime. Prime and target were presented at the center of the screen. Targets were visible for 3000 ms, or until the participant made a button-press response. The inter-trial interval was 1000 ms. Items were presented in black 16-point Courier font, on a white background. Each participant was presented with a different random order of prime-target pairs.

Participants made a lexical decision for each target by pressing the right button (comma ‘,’ key) for words, and the left button (‘c’ key) for nonwords. They responded to 20 practice trials before the experimental session. There was a short pause midway through the session.

Finally, to obtain a measure of participants’ spelling skills, after completing the lexical decision task, participants also completed a spelling from dictation test. A native English speaker dictated each of the fifteen words one-at-a-time. Each word’s primary definition was given. Participants had unlimited time to respond, and all participants received the words in the same order.

### 4.4 Results

#### 4.4.1 Analysis with classical lexical-distributional predictors

The overall accuracy of responses was high. For the reaction time (RT) analyses, we excluded 19% of the data from the full dataset, which included all incorrect trials (7.94%), as well as missing data points, latencies shorter than 100 ms and a few outlier data points (which were diagnosed with a quantile-quantile plot; additional 0.25%). There was one item that was identified as misspelled in post-experiment preliminary analysis. It too was removed (2.38%). One additional item was removed due to missing lexical-distributional predictors (2.38%), and three more items were missing discrimination-based predictors (6.05% of the data). Full details on the stimuli used in the analyses, together with reaction times and predictors, are available at the Mind Research Repository at http://openscience.uni-leipzig.de, paper package Milin2017_1.0.

As in the case of our analysis of lexical decision latencies from the English Lexicon Project [44], we applied a negative reciprocal transformation (−1000/*RT*) to the remaining RTs.

The dataset (with 5783 data points and a mean reaction time latency 655.91 ms (Median = 608 ms; SD = 189.77 ms) was submitted to an analysis with the generalized additive mixed model (gamm), using the bam function from the mgcv package [86] in the R statistical environment [103]. (For a concise introduction to gams, see [104]). The models reported here are the result of exploratory data analyses, with incremental theory-driven model building. We used fREML as the method for smoothing parameter estimation, and also made use of discretization of covariates by setting the discrete option to TRUE, which offers considerably enhanced computational efficiency.

A gamm with random intercepts for target and prime, by-subject factor smooths for trial, by-subject random slopes for pc2; with spelling, pc1 and pc2 as covariates; sub-experiment as control factor; and prime type as factor of critical interest, did not provide good support for prime type. The exhaustive and partially decomposable primes did not differ significantly in the mean at *α* = 0.05, and Wald’s test for parameter contrasts ($\chi_{\left(1\right)}^{2}=0.186$; *p* = 0.666) did not support a difference between the group means of the exhaustive and partial primes. Counter to the prediction of morpho-orthographic theory, response behavior to targets presented after corner and cornea type primes did not differ. Solid support for prime as random-effect factor (*p* = 0.0013) indicates that participants were sensitive to the presence of the primes. In order to maximize power for a group-wise priming effect, we pooled the exhaustively and partially decomposable prime levels, and proceeded with a two-level factor (unrelated primes vs. decomposable primes). Table 8 presents the statistics for the partial effects in this second gamm.

**Table 8:** Generalized additive mixed model fitted to the lexical decision latencies of experiment 1a–b using lexical-distributional predictors.

A. parametric coefficients	Estimate	Std. Error	t-value	p-value
Intercept	-1.5060	0.0378	−39.8909	< 0.0001
Experiment = 1a	-0.0543	0.0278	-1.9539	0.0508
PrimeType = decomposable	-0.0177	0.0094	-1.8801	0.0601
Spelling Proficiency	-0.1442	0.0591	-2.4390	0.0148
B. smooth terms	edf	Ref.df	F-value	p-value
tensor product smooth PC1 by PC2	4.7244	4.7768	8.2846	< 0.0001
by-Participant factor smooths for Trial	393.6075	1527.0000	2.0833	< 0.0001
by-Previous-Target random intercepts	43.0657	139.0000	0.4539	0.0003
by-Prime random intercepts	28.0480	106.0000	0.4736	0.0014
by-Target random intercepts	27.9050	33.0000	16.8749	< 0.0001

A: parametric coefficients; B: effective degrees of freedom (edf), reference degrees of freedom (Ref.df), F and p values for the non-linear terms, tensor products and random effects. AIC = 1244.3; fREML = 886.4; R-sq. = 0.492.

Even the group difference in response time between decomposable primes and unrelated primes did not receive solid support (*p* = 0.0601), replicating earlier results reported by [41]. It is only when the prime random effect factor is excluded from the model specification, that the difference between the two priming conditions (decomposable vs. unrelated) receives improved statistical support (*p* = 0.018). Although this removal brings the results in line with the experiments reported and discussed in [31, 36], it comes at the cost of a significant reduction in model fit (difference in aic: 20.03, evidence ratio exp^20.03/2^ = 22,359.4; difference in ML: *χ*^2^(1) = 4.708; *p* = 0.002). Apparently, a major part of the variance that can be explained by the experimental prime manipulation originates from random variation among the prime words.

The model summary in Table 8 further shows that participants may have responded somewhat more slowly in Experiment 1b (*p* = 0.0508). This difference can be attributed to the manipulation of form similar vs. dissimilar word prime—nonword target pairs: when 60% were similar in form as in Experiment 1a decision latencies were slightly faster than when all pairs were dissimilar as in Experiment 1b. Consistent with previous findings of [105, 106] and [107], this suggests that participants form expectations based on form similarity of the target with the prime. In particular, it seems that they can adapt to confounds within an experimental design, and rely more or less on prime-target form similarity, with their responses reflecting the degree to which form similarity was informative about target lexicality [106].

Table 8 also indicates that facilitation increased with participants’ spelling proficiency (*p* = 0.0148). We tested for an interaction with prime manipulation, but no evidence for such an interaction was found.

**Fig 7:**
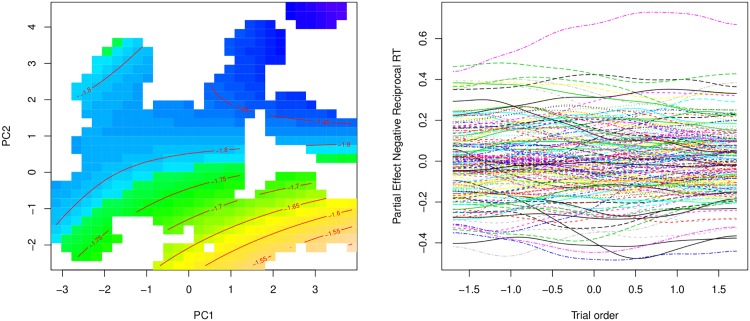
Nonlinear effects in experiments 1a and 1b. Left panel: tensor product smooth for the nonlinear interaction of the principal component for neighborhood similarity structure (pc1) and frequency of occurrence (pc2) in Experiment 1. Darker colors indicate shorter response latencies. Right panel: Partial effect of the by-participant random smooths for Trial. Each curve represents a different participant.

The statistics in part B of Table 8 document the nonlinear interactions and the random-effects structure. The tensor product smooth for the interaction of pc1 and pc2 (*p* < 0.0001) is depicted in Fig 7 (left panel), which projects the fitted regression surface onto the pc1-pc2 plane. Blue colors indicate short response latencies. As latencies increase, colors change from blue to green and finally yellow. Contour lines connect points on the surface with the same height, i.e., the same response latencies. The numbers on the contour lines are on the −1000/*RT* scale. The contour plot reveals that response latencies are long when pc1 assumes larger values while at the same time pc2 has lower values. For low values of pc1, there is hardly any effect of pc2, but for large values of pc1, the effect of pc2 is strong, with a large downward sloping gradient. Conversely, there is little effect of pc1 for large values of pc2, but for small values of pc2, pc1 shows a steep upward sloping gradient. In other words, processing times are more prolonged for lower-frequency words (negative values on pc2) that have fewer neighbors and neighbors that are located at greater distances (positive values of pc1).

The second row of part B of Table 8 reports the statistics for the by-participant factor smooths for Trial (*p* < 0.0001), depicted in the right panel of Fig 7. Each curve in this figure represents a subject, and graphs how the subject’s performance slowly changes over the the experiment as a consequence of learning, fatigue, or changes in attention (see also [108] and [109] for detailed discussion of nonlinear random effects). The plot shows, for instance, that the subject who ended up being one of the fastest responders started out with a response speed close to average, increased response speed until about two-thirds through the experiment, and then could not maintain this high level of performance and slowed down (lower black solid line). One of the slowest responders (upper dashed-dotted pink line) showed the reversed pattern.

With respect to inter-trial dependencies, the gamm also supported random intercepts for the preceding target in the experimental list (*p* = 0.0003). These random intercepts again are in line with the hypothesis of [106] and [105] that the magnitude of facilitation in a priming experiment may depend on the properties of preceding items.

To test the morpho-orthographic claim for semantically blind segmentation, prime-target pairs for this experiment were selected to be semantically unrelated. To verify that semantic confounds are not an issue, we also explicitly tested semantic similarity effects in our model. We probed both the Latent Semantic Analysis [18] and HiDEx [21] similarity measures for prime and target pairs. As neither of the two measures reached statistical significance (*p* > 0.05), we conclude that the semantic relationship between primes and targets did not demonstrably influence decision latencies in our experiments.

Experiment 1 shows that the prediction of morpho-orthographic segmentation theory [31, 36]—greater facilitation for primes that are exhaustively decomposable into morphemes compared to primes that are only partially decomposable—fails to receive support: We did not detect any such difference. Instead, we observed a weak priming effect for targets with substantial string overlap with their primes, as compared to the unrelated condition, irrespective of whether the non-overlapping part of the string is a suffix of English. Furthermore, the strong support for prime as random-effect factor indicates that most of the variance due to the priming manipulation originates from random between-prime variation.

We next proceed to complement the statistical analysis with a gamm with predictors grounded in learning theory. Central questions motivating this second analysis are, first, whether the learning-based predictors provide a tighter fit to the reaction times, and whether learning-based measures, if successful, shed further light on the question of morpho-orthographic segmentation.

#### 4.4.2 Analysis with discrimination-based predictors

**Fig 8:**
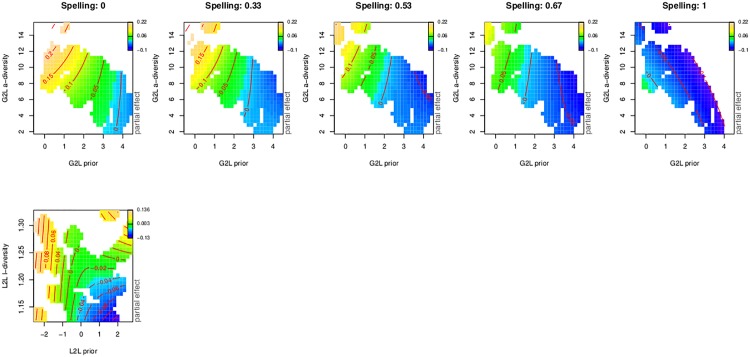
Tensor product regression smooths for experiment 1, discrimination-based predictors. Upper panels: the three-way interaction of G2L prior, G2L a-diversity, and Spelling. Lower panels: the two-way interaction of L2L prior by L2L I-diversity.

Table 9 and Fig 8 report the gamm fitted to the primed lexical decision times using learning-based predictors (using as before fREML as smoothing parameter estimation method, and setting the option discrete to TRUE for storage and computational efficiency reasons). In this analysis, support for an effect of prime type remained marginal (*p* = 0.0586), and the difference between Experiment 1a and 1b was again barely significant (*p* = 0.0497).

**Table 9:** Generalized additive model for the response latencies in experiment 1, using discrimination-based predictors.

A. parametric coefficients	Estimate	Std. Error	t-value	p-value
Intercept	-1.5789	0.0278	-56.7847	0.0000
Experiment = 1a	-0.0546	0.0278	-1.9627	0.0497
PrimeType = decomposable	-0.0178	0.0094	-1.8919	0.0586
B. smooth terms	edf	Ref.df	F-value	p-value
tensor product smooth G2L prior by G2L a-diversity by Spelling	11.1763	13.6993	2.2615	0.0054
tensor product smooth L2L prior by L2L l-diversity	4.6038	4.6664	3.4792	0.0047
by-Target random intercepts	24.5204	30.0000	12.2987	0.0000
by-Prime random intercepts	28.2487	103.0000	0.4863	0.0010
by-Previous-Target random intercepts	43.4725	139.0000	0.4600	0.0002
by-Participant factor smooths for Trial	392.9230	1527.0000	2.0703	0.0000

AIC = 1237.009; fREML = 877.48; R-sq. = 0.494.

The two prior by diversity interactions reported above for unprimed lexical decision data from the British Lexicon Project re-appear in the present gamm.

The interaction of L2L prior by L2L l-diversity (lower left panel) resembles the interaction of these predictors for the young subjects in unprimed lexical decision (see Fig 6, third panel on the top row). In both cases, we find slowing of responses for increasing lexome diversity across all values of the L2L prior, and a U-shaped effect of L2L prior for higher values of L2L l-diversity.

As in the unprimed lexical decision data, the combination of a low G2L a-diversity and a high G2L prior affords short response latencies, whereas long latencies arise for low values of the G2L prior and high values of the G2L a-diversity (upper left panels of Fig 8, see also the upper left panel of Fig 6). Interestingly, this interaction of G2L prior by G2L activation diversity was further modulated by spelling proficiency. This three-way interaction appears in the top panels of Fig 8, which present the regression surface of the G2L predictors for characteristic values of Spelling. As spelling proficiency increases, the effect size of G2L prior decreases. At the same time, the slowing of responses as a consequence of G2L activation diversity decreases as well, and for highly-proficient spellers even reverses into slight facilitation. As a consequence, for the most highly skilled participants, lexicality decisions are determined primarily by the L2L prior and diversity measures.

The interaction of semantic typicality and semantic density observed above for unprimed lexical decision did not emerge as statistically significant in the masked priming task.

The model with discrimination-based predictors outperforms the model with lexical-distributional predictors in terms of goodness of fit. The fREML score for the discriminative model (886.39) is lower by 8.91 units than that of the model with lexical-distributional predictors (877.48), and the investment of 9 additional degrees of freedom is evaluated as providing a superior fit by a chi-squared test (*p* = 0.037), thus providing further support for the importance of discrimination learning for lexical processing. The aic values for the models, 1244.3 for the model with classical predictors, and 1237.0 for the model with learning-based predictors, also favor the latter with an evidence ratio of *e*^(1244.3−1237)/2^ = 38.5. The squared correlation of fitted and observed (inverse transformed) RTs is also greater for the latter model (0.492 versus 0.494). Here we note that a model with only random effect factors and subexperiment as factorial predictor also has as squared correlation 0.492, which shows that only the model with learning-based predictors succeeds in moving beyond subject and experimental variance.

The absence of a robust main effect of prime in both the analysis with lexical-distributional predictors and in the analysis with discrimination-based predictors provides new insights into how a particular word prime can alter the processing of the following target word. The statistical support for by-prime random intercepts clearly indicates that individual prime words do affect lexical processing across participants. However, the factorial prime manipulation does not appear to be effective over and above the effects of the individual primes, as indicated by the strong support for by-prime random intercepts. What we suspect may be at issue here is the abundance of other words that occur as substrings of primes and targets. Above, we already mentioned embedded words such as *urn* in the prime *spurn* or *rip* in the prime *triplet*, and to this we can add many examples of embedded words in the targets (e.g., *as* in *ask*; *and* in *band, sand*; *at* in *bat, rat*; *it* in *bit, sit*; *ill* in *fill*; *in* in *fin, pin*; *am* in *ham*; *as* in *past*; *ink* in *pink*; *car* in *scar*; *tag* in *stag*; *act* in *tact*; *tar* in *tart*; *ten* in *tend*; *ex* in *text*). We know from the work of [93] that such embedded words (and also embedding words) are co-activated and influence processing (for the lexical decision taks, see [110]). However, experiments addressing morpho-orthographic segmentation with factorial designs are severely challenged when it comes to matching also for embedded (and embedding) words.

In the discrimination framework, by contrast, the co-activation of embedded and embedding words is taken into account through the activation diversity measure. This measure quantifies the co-activation across the lexicon that arises as a consequence of shared letter trigrams. We think that subliminally presented primes function in a very similar way as do embedded words. The inhibitory effect of our activation diversity measure suggests that primes are straightforward competitors that cause interference, slowing target responses just as other co-activated words do.

The perspective on priming that we are advocating is consistent with that of [111], who, within a Bayesian framework, argue that (masked) primes disrupt normal target word processing. It follows that when the baseline for comparison is not given by priming with unrelated primes, but instead by priming with identity primes, results should reverse. Specifically, with this new baseline, related prime-target pairs may now be expected to elicit longer reaction times instead of shorter reaction times. It is conceivable that, with less interference from unrelated primes, word pairs with exhaustively decomposable primes will have a processing benefit compared to word pairs with non-exhaustively decomposable primes. Changing baseline would therefore maximize the opportunity for a morpho-orthographic effect to emerge. This predictions is put to the test in Experiment 2.

## 5 Experiment 2: Primed lexical decision with low interference

In Experiment 2, we replaced the unrelated priming condition by an identity condition in a second attempt to find evidence that a true affix, in addition to a repeated stem, is sufficient to produce morpho-orthographic facilitation [31]. Since unrelated primes in Experiment 1 were matched in length with morphologically related primes, identity primes in Experiment 2 were also matched in length, by adding the appropriate number of dollar signs ($) after the stem (for example, PAST$, FROL$ $). In Experiment 2 word-nonword pairs were constructed so that, as in Experiment 1a, 60% of all pairs were similar in form. In all other relevant aspects as well, experimental items matched those in Experiments 1a and 1b.

Participants in Experiment 2 were again students from The University at Albany, State University of New York. They partially fulfilled the introductory psychology course requirements. The sample consisted of 60 students, native English speakers, with normal or corrected sight and no reading or speech disorders. None had participated in Experiment 1a or 1b.

The effect of spelling proficiency observed for Experiment 1 warrants further investigation, given the claims of [94], [95, 96], and [43, 97] that participants’ spelling proficiency moderates the effect of form similarity, and, to a lesser extent, the effect of semantic similarity in morphological priming. Andrews and colleagues suggest that the interaction of prime type with spelling skill is systematic and can be linked to differential preference for form and semantic properties of words. From this perspective, readers would differ in the precision of their orthographic representations, as measured by spelling proficiency, which would then predict the amount of morpho-orthographic facilitation in the lexical decision task.

In order to further explore the potential relevance of individual differences, participants in Experiment 2 performed a vocabulary test in addition to the spelling proficiency test as administrated in Experiment 1a and 1b. The procedure was adopted from [94]. There were 30 items, consisting of a target word and five alternatives for its meaning, which could be either a word or a short phrase. Participants’ task was to select the closest match to a target word. Their score was the number of correct selections out of 30. The correlation between the two verbal ability scores, the spelling proficiency and the vocabulary score, was moderate (*r* = 0.484; *t* = 4.182; *df* = 57; *p* < 0.001). Contrary to [94], we were not able to find participants with scores high on one and low on the other verbal ability test.

Apparatus, design and procedure were the same as in Experiments 1a and 1b.

### 5.1 Results

#### 5.1.1 Analysis with lexical-distributional predictors

For the analysis of the reaction time data, all incorrect trials were removed (7.82% of the data), as were time-outs and extreme outliers (jointly, 1.38% of the remaining data). To match the item set across all experiments the misspelled item identified in Experiments 1a and 1b was excluded again (2.38%), as well as the items missing lexical-distributional predictors (2.38% of the data) or discrimination-based predictors (6.0% of the data). In total, 20% of the data points were removed. As in previous analyses here too response latencies were transformed into inverse reciprocal values (−1000/*RT*).

The 2017 valid response latencies, which had a grand mean of 664.34 ms (SD = 214.6 ms, median = 611 ms), were entered into a gamm analysis, using, as for Experiment 1, estimation by fREML and discretization.

Replicating Experiment 1, the crucial contrast between partially and exhaustively morphologically decomposable primes was not significant (Wald test, *χ*^2^(1) = 0.212; *p* = 0.645, coefficients 0.0426 and 0.0506 with std. errors 0.0181 and 0.0182 respectively). We therefore again pooled the exhaustively and partially decomposable prime levels, and proceeded with a two-level factor (identical vs. decomposable) for prime type. Table 10 presents a summary of the resulting generalized additive mixed model.

**Table 10:** Generalized additive mixed model fitted to the lexical decision latencies of experiment 2 using classical lexical-distributional predictors.

A. parametric coefficients	Estimate	Std. Error	t-value	p-value
Intercept	-1.7086	0.0391	-43.7313	< 0.0001
PrimeType = decomposable	0.0466	0.0156	2.9907	0.0028
B. smooth terms	edf	Ref.df	F-value	p-value
tensor product smooth spelling by vocabulary	4.4080	4.4872	2.5166	0.0425
tensor product smooth PC1 by PC2	4.9225	5.0632	4.8111	0.0003
by-Participant random slopes for PC2	19.4850	58.0000	0.5139	0.0058
by-Participant factor smooths for Trial	155.0738	527.0000	1.6872	< 0.0001
by-Previous-Target random intercepts	27.6669	139.0000	0.2548	0.0173
by-Prime random intercepts	20.8703	106.0000	0.2903	0.0213
by-Target random intercepts	23.4921	33.0000	4.4773	< 0.0001

A: parametric coefficients; B: effective degrees of freedom (edf), reference degrees of freedom (Ref.df), F and p values for the non-linear terms, tensor products and random effects. AIC = 726.0; fREML = 474.8; R-sq. = 0.509.

As expected, target words preceeded by decomposable primes elicited longer reaction times than the identity prime control condition (contrast coefficient 0.0466, std. error 0.0156). In contrast to Experiment 1, the effect of prime type now received good statistical support (*p* = 0.0028).

**Fig 9:**
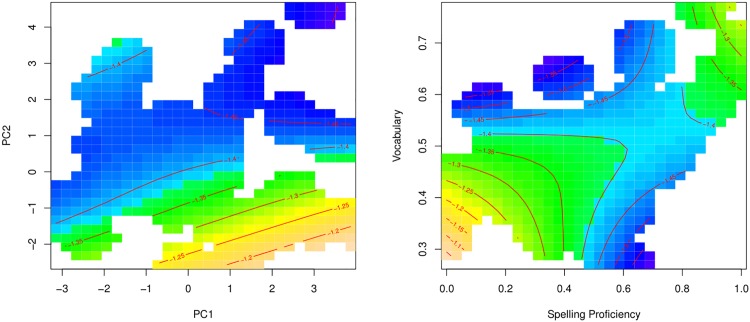
Tensor product regression smooths for experiments 2, classical lexical-distributional predictors. Left panel: tensor product smooth for the nonlinear interaction of the principal component for neighborhood similarity structure (pc1) and frequency of occurrence (pc2). Darker colors indicate shorter response latencies. Right panel: tensor product smooth for the nonlinear interaction of individual differences in spelling proficiency and vocabulary.

In addition to the effect of prime type, a tensor product smooth for the nonlinear interaction of PC1 (neighborhood structure) and PC2 (frequency of occurrence) was supported (*p* = 0.0003). This interaction closely resembled the interaction present in Experiment 1 (compare the two left panels of Figs 7 and 9). Likewise, a similar random-effects structure emerged, including random intercepts for prime. New is an interaction of vocabulary by spelling proficiency, visualized in the right panel of Fig 9. In Experiment 1, the effect of individual differences was simply linear and indicated shorter response latencies for participants with higher spelling proficiency. Here we see that this effect is further modulated by the measure of vocabulary. We find a cross-over interaction such that the effect of spelling proficiency reverses for the highest values of the other predictor. When spelling and vocabulary are both low or both high, then longer lexicality decision times are expected.

Since the regression surface in the right panel of Fig 9 closely resembles the hyperbolic plane of a straightforward multiplicative interaction, we also considered a model with a linear interaction of vocabulary and spelling proficiency. This model (not shown) did not require main effects, but supported the product of the two measures as predictor, and gave rise to an equivalent goodness of fit.

Comparing Experiments 1 and 2, we note that responses after the unrelated primes in Experiment 1 were slower, whereas after the identity primes in Experiment 2 they were faster, as expected. Hence, it is clear that priming was in fact occurring in both experiments. This conclusion is further supported by the significant contributions of the random effect of prime to the model fits for both experiments.

Exchanging unrelated primes for identity primes resulted in substantially improved support for a main effect of Prime Type. However, even though we sought to maximize the opportunity for a differential effect of partial and exhaustively decomposable primes, no such difference was supported.

#### 5.1.2 Analysis with discriminative predictors

**Fig 10:**
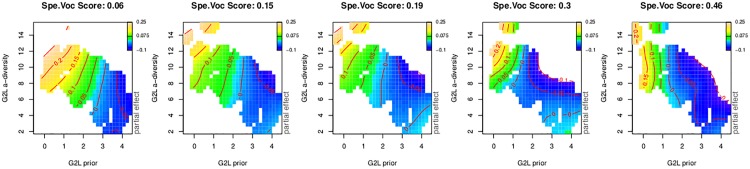
Tensor product regression smooth for the three-way interaction of G2L prior, G2L a-diversity and the spelling-vocabulary product score in Experiment 2.

Table 11 and Fig 10 present the generalized additive mixed model fitted to the response latencies of Experiment 2, using predictors grounded in discrimination learning. The freml score for the discriminative model is lower by 10.3 units than that of the model with lexical-distributional predictors (474.8 vs. 464.5, respectively), and simpler at the same time (1 degree of freedom less), again confirming the appropriateness of discrimination learning to describe lexical processing. As indicated by the evidence ratio (*e*^(725−723)/2^ = 2.72), the improvement in model fit provided by the learning-based predictors is more modest for this experiment than for Experiment 1.

**Table 11:** Generalized additive model for the response latencies in experiment 2 using discrimination-based predictors.

A. parametric coefficients	Estimate	Std. Error	t-value	p-value
Intercept	-1.6726	0.0335	-49.9040	< 0.0001
PrimeType = decomposable	0.0475	0.0155	3.0527	0.0023
B. smooth terms	edf	Ref.df	F-value	p-value
te(G2L prior by G2L a-diversity by Spe.Voc)	28.9285	40.7838	1.5446	0.0156
by-Target random intercepts	23.4973	33.0000	3.8050	< 0.0001
by-Prime random intercepts	20.8670	106.0000	0.2841	0.0204
by-Previous-Target random intercepts	27.9795	139.0000	0.2559	0.0147
by-Participant factor smooths for Trial	156.9850	529.0000	1.9029	< 0.0001

AIC = 722.5; fREML = 464.5; R-sq. = 0.511.

The random-effects structure of this model remained much the same as in the analysis with lexical-distributional predictors. The effect of prime type remained well supported (*p* = 0.0025), with the same positive sign as in the analysis with classic predictors.

Different from the preceding analysis is a significant interaction of G2L prior, G2L activation diversity, and the spelling and vocabulary measures. In order to avoid a four-way tensor product, and given the support for a multiplicative interaction of spelling proficiency and vocabulary (see the right panel of Fig 9), we opted for including as predictor the by-subject product scores of spelling proficiency and vocabulary (henceforth the Spe.Voc Score). A three-way interaction of G2L prior, G2L activation diversity, and Spe.Voc. Score was well supported statistically, and is shown in Fig 10. The general pattern is very similar to the one observed for Experiment 1 (Fig 8, top panels), in that with increasing spelling proficiency (and now also increasing vocabulary), reaction times become shorter. As before, G2L activation diversity revealed the expected inhibitory effect. However, unlike for Experiment 1, this interaction did not become progressively less important with increasing proficiency. Even for the subjects with the greatest Spe.Voc scores, both G2L predictors remained effective. By contrast, the interaction of the L2L measures, that was well supported for Experiment 1, was not predictive for Experiment 2.

It is evident that changing the nature of the control condition had profound consequences for participants’ performance. Experiment 1 confronted subjects with many trials presenting primes that mismatched their targets across the board. Proficient spellers are challenged by the inconsistent and uncooperative visual input they receive, and stop basing their decisions on the well-learned G2L sources of information (i.e., G2L prior and G2L activation diversity), which are discriminatively sufficient in felicitous reading contexts (for the adverse consequences of missfixations, see [74]), and fall back instead on an alternative source of lexical knowledge, approximated here by the L2L prior and the diversity of the target’s semantic vector. Under the adverse circumstances of Experiment 1, the lower the spelling proficiency of a participant, the more she or he will rely on the orthographic input. Nonetheless, in Experiment 1, even the least proficient spellers also use the L2L prior and the diversity of the semantic vectors.

Replacing unrelated primes by identity primes renders the visual input much more consistent and reliable. Reliance on the L2L prior and the L2L l-diversity is no longer necessary, and decisions are now based solely on the diversity of the activation vector, in interaction with the G2L prior. For subjects with greater spelling proficiency and vocabulary, the effect of the G2L prior is much stronger compared to the effect of activation diversity, whereas for the least proficient subjects, the activation diversity has a much stronger effect that disappears only for the largest priors. Thus, the present results captures proficiency in terms of having available better-tuned grapheme-to-lexome priors, which afford the reader considerable independence from the diversity of lexomes activated by the orthographic input, and the concomitant uncertainty.

## 6 General discussion

This study examined the predictions of morpho-orthographic segmentation theory under rigorous testing conditions, using both classical lexical-distributional and learning-based predictors. Counter to the predictions of the morpho-orthographic account, forward masked form-similar prime-target pairs with (corner-corn) and without (cornea-corn) exhaustively decomposable primes failed to produce differential effects (see also [40] for similar results).

Furthermore, a rigorous application of the simplest-possible error-driven learning rule [17] to big textual data (training on corpora of 0.1 and 1.1 billion words) resulted in a series of discrimination-based measures which, when tested against unprimed and primed lexical decision, outperformed classical lexical-distributional measures such as counts of frequency of occurrence and counts of lexical neighbors.

The learning-based measures capture aspects of the continuous tug of war between cues for outcomes, both at the level of sublexical orthographic features and at the level of semantic vector spaces. We note here that these measures do not constitute a mechanistic model for lexical decision making. A mechanistic computational theory will not be best realized in terms of a single ‘black box’ learning model. Rather, a large and growing body of research indicates that response learning and response selection are, in the limit, handled by different components within the brain’s architecture. Response learning seems to occur largely within the temporal and parietal lobes, whereas response selection seems to be predominantly a function of the frontal lobes [112, 113]. We harnessed the power of the generalized additive model to gauge the result of the interactions of these components and their consequences for making lexicality decisions.

The perspective on the masked priming manipulation that emerges from this study is a pessimistic one. Once the prime is taken into account as a random-effect factor, the evidence for a main effect of priming in Experiment 1 did not reach significance at *α* = 0.05. In Experiment 2, the significant main effect of priming indicated no more than that responses where slower when a ‘real’ prime, different from rather than identical to the target, was present. That response execution is slowed when the visual input is variable compared to when it is consistent is not particularly surprising. All that remains to conclude about the priming manipulation is that non-identical primes constitute a lexical source of random noise. This pessimistic conclusion will not, we hope, generalize to other masked priming experiments, but it certainly seems worth investigating how well results stand up to inclusion of the prime as random-effect factor.

With respect to the theory of morpho-orthographic segmentation, a question left unanswered by this theory is why putative form units for morphemes would exist. The theory takes as given that they do indeed exist, and proceeds to argue, on the basis of experimental evidence, that their existence can be detected with the help of masked-priming. We have shown that experimental evidence evaporates under strict experimental and statistical testing conditions. This result casts doubt on the existence of morpho-orthographic morphemes. In fact, there are good reasons for doubting that such representations are learnable.

First, the token frequencies of pseudo-suffixed words are large, whereas the token frequencies of morphologically complex words tend to be small. As a consequence, many of the tokens with a putative suffix are not morphologically decomposable, which renders an obligatory decomposition procedure computationally inefficient.

Second, most derived words have shades of meaning that are not a-priori predictable from their constituents. It is therefore inefficient for a system to split a derived word into its parts: The interpretation of that word cannot be obtained by means of a compositional semantic operation, and is available only by putting the parts back together again. It is noteworthy that if a suffix carries an unambiguous and constant semantic function, as found for instance for some inflectional suffixes in agglutinating languages such as Turkish, then in a discrimination network a strong connection will arise between the letter n-grams of that suffix and its grammatical lexome, effectively resulting in a Bloomfieldian morpheme (a unit combining form and meaning). In contrast, for English *-er*, the large numbers of pseudo-suffixed words as well as the fractionation of its semantics will stand in the way of the development of strong assocations from its letter n-grams to lexomes. In this case, units resembling the agglutinative morpheme are unlikely to come into existence.

Third, [93] have shown that embedded words that are not morphological constituents, such as *hat* in *that*, nevertheless activate their semantics. Longer orthographic strings that are not fully supported by the visual input (e.g., *corner* when *corn* is read) are also co-activated (see also for this phenomenon in the lexical decision task [110]). It follows from this research that when words such as *corner* or *brothel* are presented as primes, the semantics of the substrings *corn* and *broth* are co-activated, irrespective of morphological status. Furthermore, all kinds of other embedded words are co-activated (e.g., *owl* in *cowl*, *and* in *bandit*, *gnat* in *stagnate*). As a consequence, the array of meanings that receive partial support from the prime will include more than just the meanings of prime and target. The G2L a-diversity measure captures important aspects of this co-activation. On its own, however, G2L a-diversity cannot do justice to the extra (albeit temporally brief) support for one specific superstring of the target—namely, the prime itself. Fortunately, this extra support can be accounted for by the posterior mode for the prime in the mixed model when prime is incorporated as random-effect factor.

In summary, there are good reasons—experimental, statistical and, ultimately, cognitive and linguistic—for not expecting an effect of morpho-orthographic priming. The absence of evidence for a morpho-orthographic effect under stringent test conditions maximizing opportunities for detecting such an effect is, of course, not evidence of absence. But the specific point we want to make here is that the absence of evidence is not at all in conflict with current insights from distributional lexical statistics, linguistic morphology, and experimental psychology, and that there are actually excellent theoretical reasons for expecting a null effect.

The measures grounded in discrimination learning that we propose in this study offer two further insights. First, under the more favorable reading conditions of Experiment 2 (identity primes as baseline) compared to Experiment 1 (unrelated primes as baseline), participants no longer rely on lexome-to-lexome measures. When primes are maximally confusing, subjects rely in part on the target’s entrenchment in the semantic network (lexome-to-lexome prior) and on low numbers of strong collocates in semantic vector space (L2L l-diversity) for optimizing response times: Words that are well entrenched and that have few strong collocates are responded to more quickly. When the mismatch between primes and targets is reduced by replacing unrelated primes by identity primes, subjects no longer resort to their collocational lexical knowledge, and base their responses solely on bottom-up information, as indexed by the G2L prior and the G2L activation diversity. (A comparison of the two priming experiments with unprimed lexical decision shows that in unprimed lexical decision measures of semantic density and semantic typicality are also predictive. One possible reason for the emergence of these two semantic measures in unprimed lexical decision is that the power of the unprimed experiment is much larger, with 1812 words instead of a mere 42 target words. Alternatively, it is conceivable that in the absence of primes messing up the visual input [111], subjects can rely more on semantic density and typicality. The present data do not allow us to choose between these two explanations.)

Second, the measures grounded in learning theory clarified one component of what it means to be a proficient speller with a large vocabulary, as highly proficient participants showed greater dependence on orthographic priors, and less interference from activation diversity. This parallels a finding in unprimed lexical decision, where we observed a similar reduction in dependence on lexome-to-lexome measures for older subjects as compared to younger subjects. Thus, the wisdom that comes with age [114] makes older subjects similar to those (young) participants in Experiment 2 who are most proficient and have the richest vocabularies.
